# Efficient and Selective Extraction of Prenylated Flavonoids from *Sophora flavescens* Using Ultrasound-Assisted Hydrophobic Ionic Liquid and Characterization of Extraction Mechanism

**DOI:** 10.3390/molecules30030500

**Published:** 2025-01-23

**Authors:** Shasha Kong, Qian Liao, Yuling Liu, Ruying Tang, Longfei Lin, Hui Li

**Affiliations:** 1 Institute of Chinese Materia Medica, China Academy of Chinese Medical Sciences, Nanxiaojie 16, Dongzhimennei Ave, Beijing 100700, China; 2 Institute of Traditional Chinese Medicine Health Industry, China Academy of Chinese Medical Sciences, Nanchang 330006, China

**Keywords:** ionic liquid, prenylated flavonoids in *Sophora flavescens*, ultrasonic-assisted extraction, anti-tumor activity, density functional theory, molecular dynamics

## Abstract

As a secondary metabolite with vigorous pharmacological activity (antitumor), prenylated flavonoids in *Sophora flavescens* (PFS) have great potential to be transformed into lead compounds. Its extraction has attracted more and more attention. Conventional organic solutions cannot selectively extract PFS and then need to carry out liquid–liquid extraction and multi-step column separation, which is time-consuming and pollutes the environment. This study found that [C_8_mim]BF_4_ had a high efficiency and selectivity for extracting PFS. Under the optimal extraction parameters, the yield of PFS was 7.38 mg/g. Based on UHPLC-Q-Orbitrap MS, 39 prenylated flavonoids were identified in PFS freeze-dried powder, which showed strong anti-tumor activity. In addition, the potential mechanism of selective extraction was analyzed in depth. [C_8_mim]BF_4_ synergy ultrasound destroys the cell wall structure, making the active components in the cell more easily exposed to the extraction solvent. The formation of more hydrogen bonds and van der Waals forces between [C_8_mim]BF_4_ and PFS accelerates the dissolution of PFS.

## 1. Introduction

*Sophora flavescens*, the dried root of the *S. flavescens* Ait plant, is a famous medicinal plant with a history dating back to the Eastern Han Dynasty (A.D. 25 to 220) [1]. Modern pharmacological studies have identified several beneficial effects of *S. flavescens*, including anti-inflammatory [2], antioxidant [3], immunomodulatory [4], hypoglycemic [5], and anti-tumor properties [6]. The chemical composition of *S. flavescens* is complex and diverse, containing alkaloids, flavonoids, phenolic acids, triterpenoid saponins, lignans, and other components [1]. Early basic research on the pharmacodynamic substances of *S. flavescens* believed that alkaloids were the key indicators for its powerful biological activity. However, with the deepening of research, more and more scholars have realized the importance of prenylated flavonoids in *S. flavescens* (PFS) in recent years. Prenylated flavonoids are a unique secondary metabolite, mainly in leguminous Chinese herbs [7]. These flavonoids have prenyl side chains attached to the flavonoid parent nucleus (C_6_-C_3_-C_6_), making them fat-soluble and enhancing their affinity to cell membranes. Consequently, they demonstrate stronger anti-inflammatory, anti-tumor, and antibacterial activities than regular flavonoid components [1,8]. Research also indicates that the anti-tumor activity of PFS is more significant than that of alkaloids [1]. Therefore, PFS has great potential to be converted into lead compounds.

Currently, the extraction of PFS primarily relies on traditional solvents like ethanol and methanol. Aside from prenylated flavonoids, the extraction solution contains numerous other compounds, including alkaloids and saponins [9]. To achieve high-purity prenylated flavonoids, the process typically involves liquid–liquid extraction, column chromatography, and other methods for further enrichment and purification [10]. However, the above methods are time consuming, labor intensive, and costly. Moreover, using many organic reagents in liquid–liquid extraction and column chromatography poses environmental and health risks due to their toxicity, volatility, and flammability. Therefore, finding a cleaner and more efficient solvent with selective extraction of target ingredients can reduce time costs and improve work efficiency.

Ionic liquids (ILs), a type of liquid molten salt, are known for their non-volatility, thermal stability, and non-flammability. It demonstrates excellent solubility to various small molecule compounds and is considered an environmentally friendly alternative to traditional organic solvents [11]. Different combinations of cations and anions make it possible to synthesize various ILs with custom properties, offering versatility for experimental needs. Although studies have indicated some toxicity to algae and microorganisms, the toxicity level of ILs is significantly lower than that of methanol and acetonitrile [12,13]. In recent years, ILs have been increasingly utilized in diverse scientific fields for separation, electrochemical analysis, and chromatographic analysis, particularly in extracting natural small-molecule active ingredients [14]. Hydrophobic ILs usually refer to ILs with hydrophobic anion groups, such as CF_3_SO_3_^−^, PF_6_^−^, and BF_4_^−^. For 1-alkyl-3-methylimidazole cations, the hydrophobicity of IL increases with the alkyl chain length. When the alkyl chain length reaches or exceeds the octyl, the IL is characterized by non-aqueous miscibility [15]. Due to the large viscosity of hydrophobic ILs and the difficulty of phase separation, there are few reports on the direct extraction of natural products based on hydrophobic ILs [16]. High temperature can overcome the high viscosity of hydrophobic ILs, which provides a possibility for extracting non-polar compounds in Chinese herbs. Recent work has shown that pure hydrophobic ILs are used to selectively extract small amounts of prenylated flavonoids from licorice [17]. However, a comprehensive analysis of the extraction mechanism and an assessment of the chemical profile of purified natural products from IL extraction still needs to be conducted.

In this study, 11 types of hydrophilic and hydrophobic ILs were developed to align with the physical properties of PFS. Following an evaluation of the extraction yields of five representative PFS compounds (kushenol I, KSI; kurarinone, KRN; sophoraflavanone G, SFG; 2′-methoxykurarinone, MKR; isokurarinone, IKR), [C_8_mim]BF_4_ was identified as the most effective solvent. On the basis of the single-factor test and response surface design, the key parameters of the extraction process, such as solvent-to-solid, extraction time, soaking time, and extraction temperature, were optimized. PFS-rich freeze-dried powder (PFS-FP) was recovered using reversed-phase solid-phase extraction (RP-SPE), and its composition was analyzed using ultra-high-performance liquid chromatography (UHPLC)-Q-Orbitrap mass spectrometry (MS). Scanning electron microscopy (SEM) and Fourier transforms infrared (FT-IR) were used to observe the surface morphology of the samples. At the same time, density functional theory (DFT) and molecular dynamics (MD) techniques were used to simulate the change in the force between the extraction solvent and the target compound during the extraction process, the extraction mechanism of the ultrasound-assisted [C_8_mim]BF_4_ (UA-[C_8_mim]BF_4_) was discussed. Finally, the anti-tumor activity of PFS on HepG2 cells was evaluated by measuring their proliferative activity and cell viability.

## 2. Results and Discussion

### 2.1. Selection of ILs

ILs typically consist of organic cations and organic or inorganic anions [18]. The structure of an IL is critical in determining its physicochemical properties and its ability to extract bioactive ingredients from natural plants. Imidazole ILs are commonly used to extract natural products such as flavonoids, polyphenols, volatile oils, and saponins [17,19,20,21,22]. These ILs can dissolve plant cell wall cellulose and facilitate the release of target compounds [20,21]. In this study, the properties of the target compound, prenylated flavonoids, and the interfering component, alkaloid, need to be considered to select an ionic liquid to extract prenylated flavonoids from *S. flavescens*. Prenylated flavonoids are hydrophobic due to the presence of prenyl side chains, while interfering flavonoids and alkaloids are hydrophilic. Following the “similarity compatibility theory”, 1-alkyl-3-methylimidazole ILs were chosen, and 11 types of hydrophilic and hydrophobic ILs (Table S1) were designed and tested by modifying the alkyl carbon chain length and anion type in order to obtain the best ILs.

**Figure 1:**
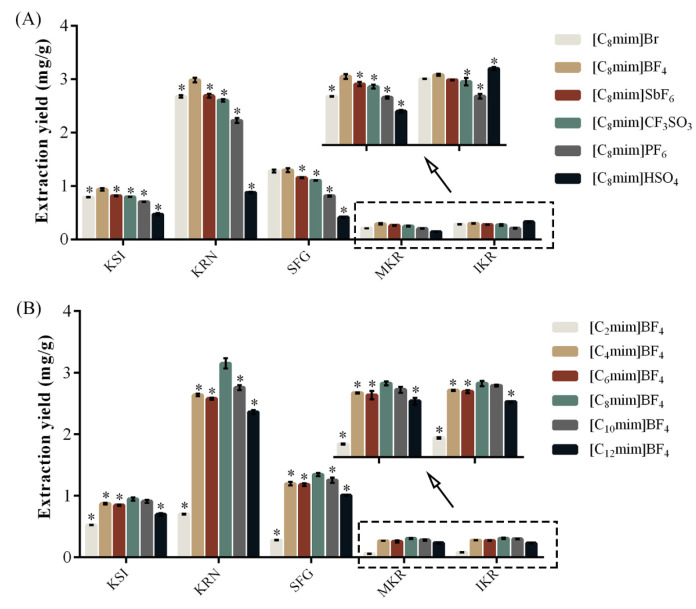
Influences of differential ionic liquids on the extraction yield of KSI, KRN, SFG, MKR, and IKR from *S*. *flavescens*. (**A**) differential anions; (**B**) differential alkyl side chain lengths of cations; *n* = 3, * *p* < 0.05 vs. [C_8_mim]BF_4_.

When using 1-octyl-3-methylimidazole as the cation, the extraction yields of prenylated flavonoids from six ILs, namely [C_8_mim]Br, [C_8_mim]PF_6_, [C_8_mim]HSO_4_, [C_8_mim]SbF_6_, [C_8_mim]CF_3_SO_3_, and [C_8_mim]BF_4_, were investigated. As shown in Figure 1A, anions significantly affect KSI, KRN, SFG, MKR, and IKR extraction yield. [C_8_mim]BF_4_ exhibited the highest extraction yield for prenylated flavonoids, indicating that BF_4_^−^ may have stronger interactions with PFS, such as hydrogen bonds, halogen bonds, and van der Waals forces [23].

The length of the alkyl chain in 1-alkyl-3-methylimidazole-type cations affects the polarity of ILs, impacting the extraction yield of prenylated flavonoids. In this study, six different ILs were created using cations with varying carbon chain lengths ([C_2_mim]^+^, [C_4_mim]^+^, [C_6_mim]^+,^ [C_8_mim]^+^, [C_10_mim]^+^, and [C_12_mim]^+^) combined with BF_4_^−^. The results revealed that the extraction yields of KSI, KRN, SFG, MKR, and IKR increased significantly as the alkyl side chain lengthened from ethyl to octyl (Figure 1B). This may be due to factors such as increased hydrophobicity, larger contact area, and stronger interaction forces between ILs and prenylated flavonoids. However, when the alkyl side chain went from octyl to dodecyl, the extraction yields decreased (Figure 1B), likely because of the higher density and viscosity of ILs [17], which affected its ability to solvate the prenylated flavonoids [22]. In conclusion, [C_8_mim]BF_4_ was found to be the most effective solvent for extracting prenylated flavonoids.

### 2.2. Optimization of Extraction Conditions by Single-Factor Experiments

**Figure 2:**
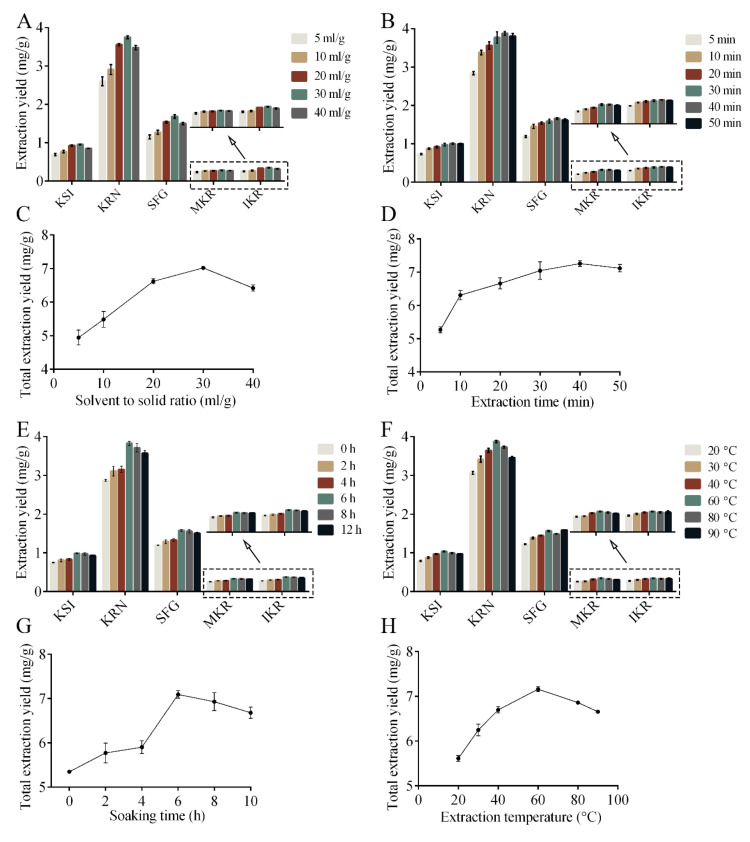
Effect of different factors on the extraction yield of PFS. Effect of solvent to solid ratio on the extraction yield of five PFS (**A**) and total PFS (**C**); Effect of extraction time on the extraction yield of five PFS (**B**) and total PFS (**D**); Effect of soaking time on the extraction yield of five PFS (**E**) and total PFS (**G**); Effect of extraction temperature on the extraction yield of five PFS (**F**) and total PFS (**H**).

As shown in Figure 2A,C, the solvent-to-solid ratio greatly influences the extraction yield of PFS. When the solvent-to-solid ratio increased in the range of 5–30 ml/g, the total extraction yield of PFS increased, indicating that the extraction was incomplete. This may be because a higher solvent-to-solid ratio leads to a larger contact area, increasing the mass transfer efficiency [17]. Further growing the solvent-to-solid ratio (30–40 mL/g), the extraction yield decreased. It is speculated that the contact area between [C_8_mim]BF_4_ and target compounds is saturated, and the large volume of extraction solvent will disperse ultrasonic energy [24], which is not conducive to ultrasonic-assisted extraction.

Extraction time greatly affects the extraction yield of PFS (Figure 2B,D). When the extraction time was increased in the range of 5–40 min, the extraction yield of PFS gradually increased, and the maximum extraction yield was reached at 40 min. Further extension of extraction time resulted in a slight decrease in the extraction yield of PFS. The results above showed that the target compound was completely extracted when the extraction time reached 40 min. However, the further extension of ultrasonic time can cause serious structural damage, inter-bubble collision, and saturation effects [25], which reduces the extraction rate of PFS after 40 min.

The extraction yield of PFS increased gradually as the soaking time extended from 0 to 6 h, reaching its maximum at 6 hours (Figure 2E,G). However, with further increases in soaking time beyond 6 hours, the extraction yields of KSI, KRN, and SFG experienced a slight decrease. This observation aligns with the findings reported by Ji et al. [17]. It is speculated that prolonged soaking may allow other components to compete with the target components to extract the solvent [26], resulting in reduced extraction yields of KSI, KRN, and SFG.

ILs have large viscosity, requiring temperature increases to lower it when using ILs [27]. Therefore, temperature may be a key factor affecting the extraction yield of PFS. As shown in Figure 2F,H, the extraction yield of PFS gradually increased when the temperature increased in the range of 20–60 °C. This could be due to the decrease in viscosity of ILs as temperature increases, along with the enhanced diffusivity of the solvent in plant cell tissue, which facilitates the release of the target compound [28]. With further increased extraction temperature, the extraction yield of KSI and KRN began to decline. This decline may be attributed to high temperatures causing alterations in the chemical structures of KSI and KRN. Consistent with previous studies [29], it was revealed that the appropriate temperature helps to increase the extraction yield of the target compound.

### 2.3. Optimization of Extraction Conditions Based on Box-Behnken Design

The Box-Behnken design (BBD) experiment was utilized to further optimize the solvent-to-solid ratio, extraction time, soaking time, and extraction temperature. Table 1 presents the independent variables and response values corresponding to the 29 experiments conducted. A quadratic polynomial stepwise regression analysis was performed on the experimental results, leading to the development of the following final mathematical model: Y = 7.32 − 0.108A − 0.083B + 0.019C − 0.029D − 0.013AB − 0.039AC + 0.138AD − 0.074BC − 0.050BD + 0.018CD − 0.218A^2^ − 0.224B^2^ − 0.156C^2^ − 0.166D^2^

As shown in Table 2, the F value is 15.03, and the *p* value is <0.0001, indicating that the fitted quadratic polynomial model contributes significantly to the PFS extraction rate [30]. The F value of the lack of fit is 1.24, and the *p* value is 0.4515 (>0.05). Since the lack of fit evaluates the model’s failure to represent experimental data other than regression, the model equation is insignificant relative to the pure error [29]. R^2^_adj_ evaluates the goodness of fit of the regression equation and represents the degree of correlation between the observed and predicted values [29]. R^2^_adj_ = 0.8888, meaning that the model can explain 88.88% of the total variation, indicating that the established model equations have universal validity and accuracy. A small coefficient of variation (C.V.% = 0.9180) indicates that the experimental results have high repeatability and reliability. Overall, the above results show that the PFS extraction yield model is very significant, and the correlation between the measured and predicted data is acceptable for any combination of the proposed variables.

*p*-values were used to evaluate the significance of each coefficient and the correlation between each variable [30]. The larger the F-value, the smaller the *p*-value, and the more significant the corresponding variable is considered. As shown in Table 2, the variable that had the greatest influence on the extraction yield of PFS was the quadratic term of extraction time (B^2^) (F = 82.26; *p* < 0.0001), followed by the quadratic term of solvent-to-solid ratio (A^2^) (F = 50.12; *p* < 0.0001) and the quadratic term of soaking time (D^2^) (F = 49.23; *p* < 0.0001). The independent variable soaking time (D) and the four interaction terms (AB, AC, BD, CD) had no significant difference (*p* > 0.05). The above results suggest that extraction time (B) is the most important factor affecting the extraction yield of PFS.

**Figure 3:**
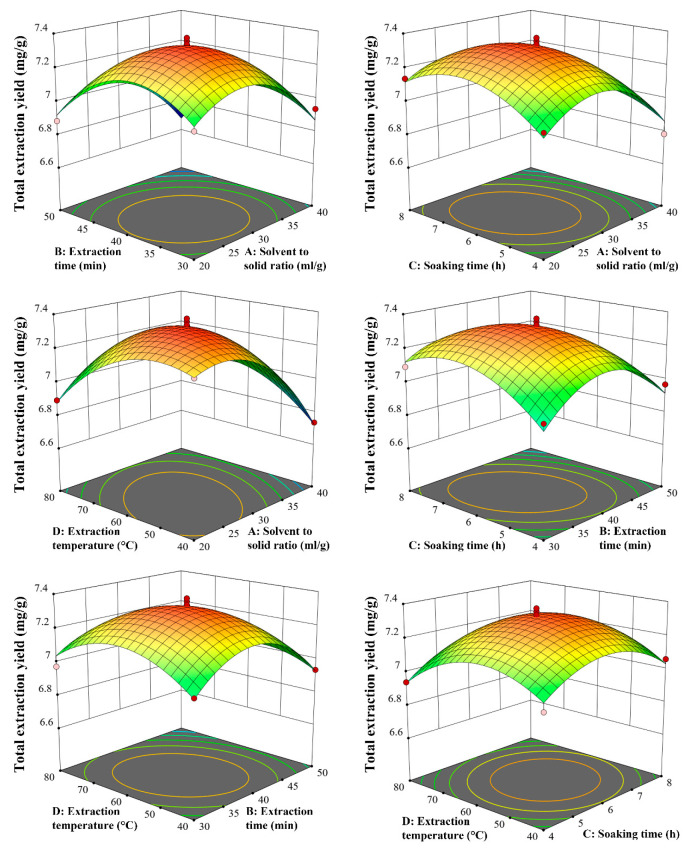
Three-dimensional response surface diagrams of the influence of different factors on the composite evaluation value.

The interactive effects of different extraction conditions on the extraction yield of PFS were visualized by a 3D response surface diagram (Figure 3) and a contour map (Figure S1). The optimal extraction parameters were as follows: the solvent-to-solid ratio was 26.88 mL/g, the extraction time was 38.22 min, the soaking time was 6.27 h, and the extraction temperature was 56.35 °C. In the verification experiment, the process parameters were adjusted as follows: solvent-to-solid ratio, 27 mL/g; Extraction time, 38 min; Soaking time, 6 h; Extraction temperature, 56 °C. A total of 6 parallel tests were conducted. The average total extraction rate of PFS was 7.38 ± 0.19 mg/g, which was not significantly different from the model prediction value, indicating that the established response surface model and polynomial equation are accurate and repeatable for the expected optimization of ultrasonic-assisted extraction of PFS.

### 2.4. Selectivity and Efficient Extraction Ability of [C_8_mim]BF_4_

**Figure 4:**
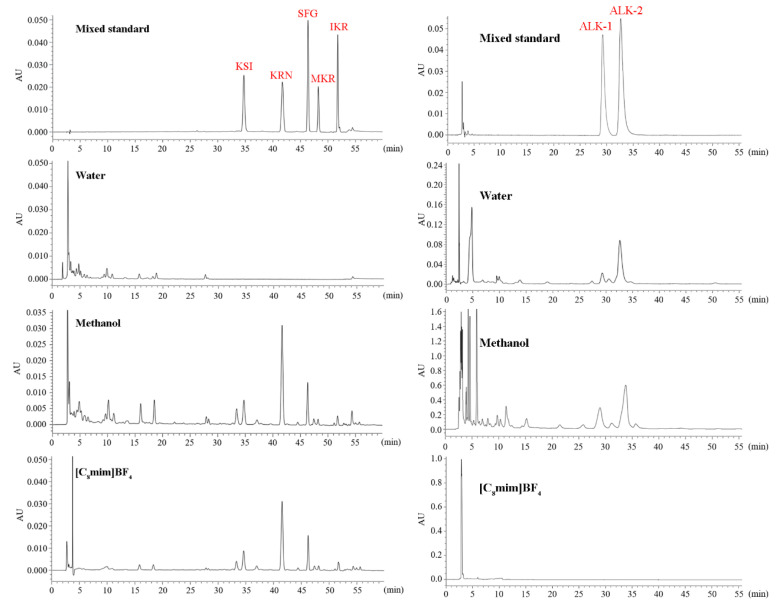
HPLC chromatograms for reference compounds (KSI, kushenol I; KRN, kurarinone; SFG, sophoraflavanone G; MKR, 2′-methoxykurarinone; IKR, isokurarinone; ALK-1, matrine; ALK-1, oxymatrine) and *S. flavescens* extraction solutions. (**Left**, a flavonoid chromatogram; 0–30 min, flavonoids; 30–55 min, prenylated flavonoids; **right**, alkaloid chromatogram).

To elucidate the targeting of [C_8_mim]BF_4_ on the extraction of PFS, we conducted ultrasonic-assisted extraction of *S. flavescens* using two conventional solvents, methanol, and water, commonly utilized for extracting active ingredients from TCM. Subsequently, we analyzed the HPLC spectra of alkaloids and prenylated flavonoids in the conventional extraction solution as well as the [C_8_mim]BF_4_ extraction solution. The results (Figure 4) demonstrate the preferential extraction of PFS by ILs, with hydrophilic components (flavonoids and alkaloids) largely absent in the extraction solution. Conversely, water displayed strong selectivity for flavonoids and alkaloids in *S. flavescens,* but did not demonstrate the ability to extract prenylated flavonoids. The conventional organic solvent methanol exhibited a strong capability to extract both hydrophilic components (alkaloids, flavonoids) and hydrophobic components (prenylated flavonoids).

Furthermore, the extraction yields of KSI, KRN, SFG, MKR, and IKR in methanol and [C_8_mim]BF_4_ were quantitatively analyzed. In the [C_8_mim]BF_4_ extraction solution, the extraction yields of KSI, KRN, SFG, MKR, and IKR were 0.95, 3.15, 1.34, 0.30, and 0.30 mg/g, respectively (Figure S2). The extraction yields in the methanol extraction solution were 0.67, 2.25, 0.93, 0.18, and 0.22 mg/g, respectively (Figure S2). [C_8_mim]BF_4_ demonstrated a stronger ability to extract PFS than methanol. ILs may disrupt the structure of the plant cell wall, potentially by increasing the cellulase activity or binding with cellulose [20]. This disruption could reduce the dissolution resistance of active ingredients, resulting in increased extraction yields of active ingredients.

### 2.5. Recovery of PFS and Recycling of ILs

The recovery of target compounds from ILs has been challenging due to their high boiling point and difficulty in evaporation. Methods such as antisolvent, recrystallization, and chromatographic separation are commonly employed for recovery [17,19]. However, in this study, due to the stability of the [C_8_mim]BF_4_-PFS extraction system, recrystallization is not feasible for PFS recovery, and no suitable antisolvent has been identified. Therefore, we utilized Oasis HLB extraction cartridges to recover PFS from the [C_8_mim]BF_4_ extraction solution. It was found that ILs could be completely eluted with 5BV 50% methanol, leaving almost no PFS in the elution solution. Subsequently, PFS was eluted using 5BV methanol without [C_8_mim]BF_4_ in the elution solution (Figure S3). The recoveries of KSI, KRN, SFG, MKR, and IKR are within the range of 62.32–85.15% (Table S4). Additionally, the chemical profiles of the PFS-rich freeze-dried powder (PFS-FP) were analyzed based on UHPLC-Q-Orbitrap MS (Supplementary Material), and a total of 39 prenylated flavonoids were identified (Table S6).

The recyclability of [C_8_mim]BF_4_ was investigated from an economic standpoint, which is crucial for large-scale production. The extraction yields of MKR and IKR decreased significantly after ILs were recovered twice (Figure S4A). KSI, KRN, and SFG extraction yields did not decrease significantly until ILs were recovered four times (Figure S4A). The extraction yield of total prenylated flavonoids decreased significantly after ILs were recovered four times (Figure S4B). The purity of ILs gradually reduced with each recovery, leading to a gradual decrease in its extraction ability. In summary, the recyclability of [C_8_mim]BF_4_ is acceptable, and it can be reused at least three times, thus helping to minimize environmental impact and production costs.

### 2.6. Extraction Mechanism Exploration

In contrast to traditional extraction solvents such as water and methanol, [C_8_mim]BF_4_ demonstrates efficient and targeted extraction of PFS. In this section, the extraction mechanism of [C_8_mim]BF_4_ was analyzed in terms of the surface changes of *S. flavescens* before and after the extraction process, as well as the simulation of interaction forces within the extraction system.

#### 2.6.1. Structural Changes After Extraction

**Figure 5:**
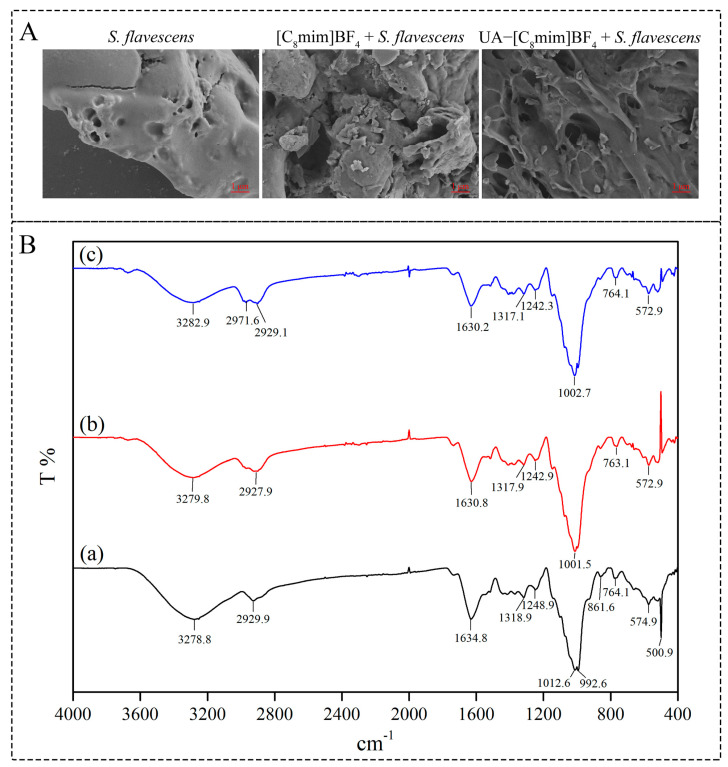
(**A**) Scanning electron micrographs of *S. flavescens* powder before and after extraction by [C_8_mim]BF_4_, UA-[C_8_mim]BF_4_; (**B**) FT-IR spectra of *S. flavescens* powder (a) before and after extraction by (b) [C_8_mim]BF_4_, (c) UA-[C_8_mim]BF_4_.

The SEM results (Figure 5A) revealed a smooth surface of the *S. flavescens* powder without any cracks before the extraction process. The surface exhibited crumpling and breakage following soaking with [C_8_mim]BF_4_. Subsequent ultrasonic-assisted [C_8_mim]BF_4_ extraction (UA-[C_8_mim]BF_4_) resulted in the appearance of distinct holes on the surface of *S. flavescens*. These findings suggest that the surface morphology of *S. flavescens* underwent significant changes after UA-[C_8_mim]BF_4_ treatment. The presence of surface holes increased the contact area between *S. flavescens* powder and ILs, thereby facilitating the diffusion of PFS and leading to an enhanced extraction yield of PFS.

The FT-IR spectra were used to observe the changes in functional groups before and after extraction. Significant changes were observed in the spectra of *S. flavescens* after immersion in [C_8_mim]BF_4_ (Figure 5B(b)) and UA-[C_8_mim]BF_4_ extraction (Figure 5B(c)). The absorption peak observed at 3279 cm^−1^ indicates the stretching vibration of O-H (ν_O-H_), which is caused by the glycosidic bond of cellulose [29]. After UA-[C_8_mim]BF_4_ extraction, the peak strength of ν_O-H_ in the sample decreased significantly, which proved that the cellulose structure was incomplete, indicating that the cell wall structure of the sample was damaged. The peak at 2930 cm^−1^ is attributed to the tensile vibration of C-O (ν_C-O_) in the cellulose [31]. After immersion in [C_8_mim]BF_4_ and UA-[C_8_mim]BF_4_ treatment, two consecutive peaks appeared at 2929 cm^−1^ and 2971 cm^−1^, which once again demonstrated the disintegration of the cellulose structure. The stretching vibration absorption band of C-O (ν_C-O_) merges into a sharp peak at 1002 cm^−1^. The above results revealed that [C_8_mim]BF_4_ synergy ultrasound destroyed the structure of cellulose, the main component of the cell wall of *S. flavescens*, and reduced the mass transfer resistance of PFS, thereby improving the extraction yield of PFS. Other researchers have confirmed the same conclusion [20,21].

#### 2.6.2. Simulation of Interaction Between PFS and Extraction Medium Based on DFT

FT-IR and SEM results showed that [C_8_mim]BF_4_ could destroy the cell wall structure of herbaceous plants. However, the interaction between PFS and [C_8_mim]BF_4_/methanol is unclear. This chapter aims to explain the interactions within the extraction system at the molecular level using DFT and MD simulations.

The structures of [C_8_mim]BF_4_, methanol and KRN were optimized, and then the interactions among them were analyzed using the electrostatic surface potential (ESP). The ESP distribution curves of [C_8_mim]BF_4_ and KRN are more similar (Figure S5), indicating a stronger interaction between [C_8_mim]BF_4_ and KRN [32]. The independent variables of the general interaction properties function (GIPF), including σ_+_^2^, σ_−_^2^, σ_tot_^2^, were used to further explore the interaction between [C_8_mim]BF_4_, methanol, and KRN. A higher σ_tot_^2^ indicates a stronger tendency for the molecule to interact electrostatically with another molecule [33]. [C_8_mim]BF_4_ has a higher σ_tot_^2^ value (Table 3), indicating that [C_8_mim]BF_4_ interacts more strongly with KRN. Specifically, BF_4_^−^ has a large σ_−_^2^ value, indicating that anions of ILs may interact strongly with KRN through their negative ESP region. The anion of [C_8_mim]BF_4_ may be the main factor for the efficient extraction of PFS. The σ_tot_^2^ value of methanol is 227.86 (kcal/mol)^2^, which is about half the value of [C_8_mim]BF_4_, indicating a weak interaction between methanol and KRN.

**Figure 6:**
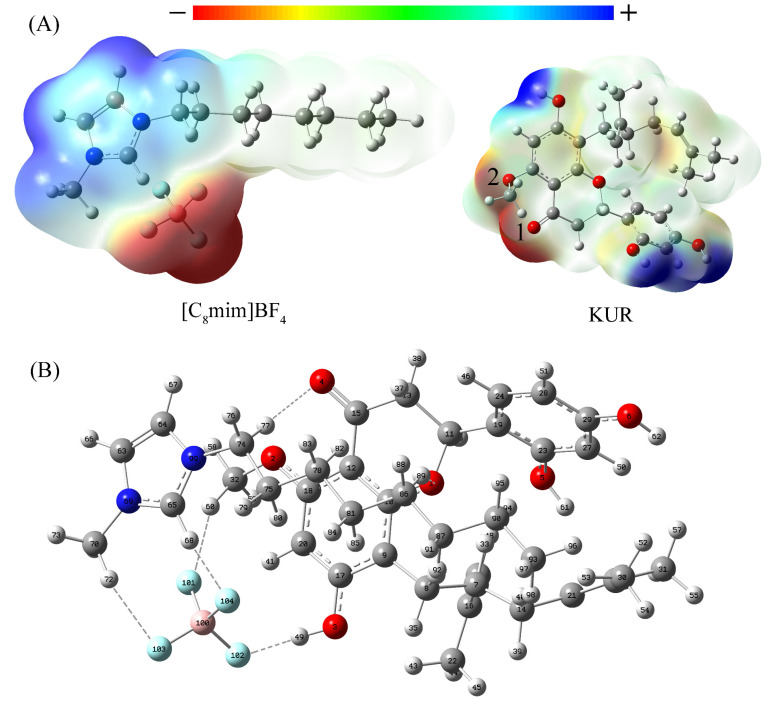
Atoms in molecules (AIM) analysis between [C_8_mim]BF_4_ and KRN. (**A**) [C_8_mim]BF_4_ and KRN electrostatic potential diagram; (**B**) Optimized complex of [C_8_mim]BF_4_-KRN (red: oxygen; gray: carbon; white: hydrogen; blue: nitrogen; green: fluorine; pink: boron).

The interaction energy between [C_8_mim]BF_4_ and KRN was analyzed. The ESP between [C_8_mim]BF4 and KRN was calculated (Figure 6A). The results indicated that the hydroxyl oxygen atom and the methoxy oxygen atom in KRN were negatively charged, with the most obvious one was between the No. 1 and No. 2 oxygen atoms. In contrast, the hydrogen atom of the hydroxyl group was positively charged. The imidazole ring of the cation exhibited very strong positive potential energy, while the end of the carbon chain showed relatively weak positive potential energy. This suggests that the imidazole ring of the cation may bind to the 1st and 2nd oxygen atoms of KRN, and the BF_4_^−^ may interact with the hydroxyl hydrogen atoms. The spatial geometry of [C_8_mim]BF_4_ and KRN was optimized according to the potential action sites. The results show that [C_8_mim]BF_4_ and KRN form intermolecular hydrogen bonds, including C(15)-O(4)···H(77)-C(74), B(100)-F(101)···H(60)-C(32), and B(100)-F(102)···H(40)-O(3) (Figure 6B). The interaction energy between [C_8_mim]BF_4_ and KRN was calculated according to Formula (1) [21].

(1) $$∆E={E_{KRN/ILs}-\left(E\right.}_{KRN}+\left.E_{ILs}\right)$$

E*_KRN/ILs_*, E*_KRN_*, and E*_ILs_* represent the [C_8_mim]BF_4_-KRN system energy, KRN, and [C_8_mim]BF_4_ energy, respectively. ΔE is calculated as −0.1 KJ/mol (Table S7), indicating that KRN can dissolve in ILs [34]. This suggests that PFS can also be dissolved in [C_8_mim]BF_4_ solution.

The strength of the non-covalent interaction between [C_8_mim]BF_4_ and KRN was analyzed using atoms in molecules (AIM). Table 4 presents the key topological parameters of the bond critical point (BCP) of the [C_8_mim]BF_4_ and KRN interaction. The values of *ρ*_BCP_ and *V*^2^*ρ*_BCP_ ranged from 0.012 to 0.041 a.u and 0.039 to 0.135, respectively, indicating a closed shell interaction between [C_8_mim]BF_4_ and KRN [34]. One of the three eigenvalues is positive, and the others are negative, demonstrating a chemical interaction between the two atoms. In addition, the value of *V*^2^*ρ*_BCP_ is greater than 0, indicating that there are non-covalent interactions, such as hydrogen bonds, van der Waals forces, ionic bonds, etc. The energy of the hydrogen bond (E_HB_) between [C_8_mim]BF_4_ and KRN molecules exceeds −14.0 kcal/mol, meaning that a weak to moderately strong hydrogen bond can form between them [35].

**Figure 7:**
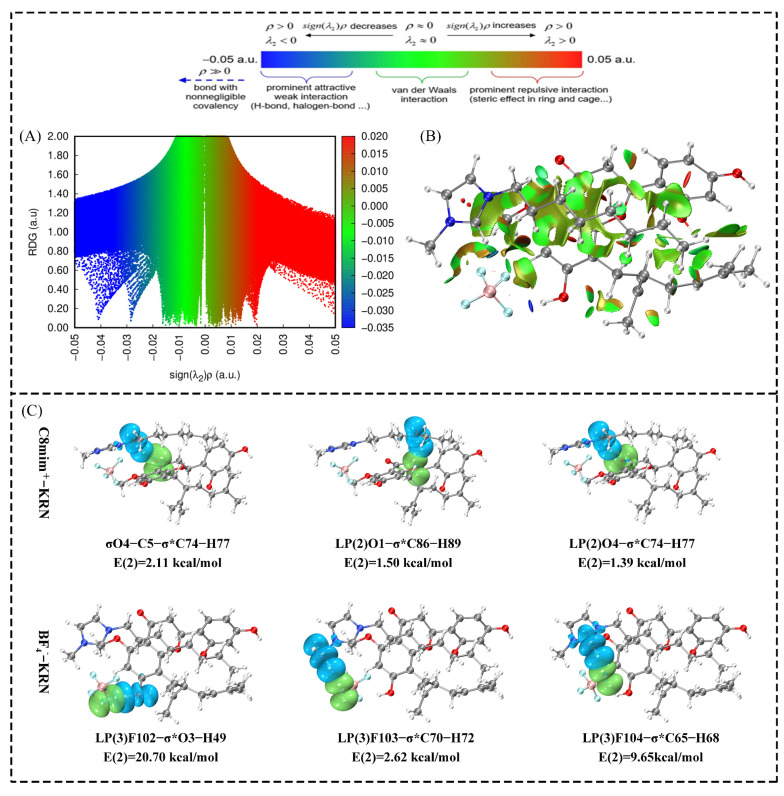
The RDG scatter plot (**A**) and sign(λ2)*ρ* mapped RDG isosurfaces (**B**) of [C_8_mim]BF_4_-KRN. (**C**) Three-dimensional overlapping images of donor-acceptor orbital interactions. (red: oxygen; gray: carbon; white: hydrogen; blue: nitrogen; green: fluorine; pink: boron; * represents conjugation in the system).

The reduced density gradient (RDG) is a method used to detect non-covalent interactions in real space based on electron density and its derivatives [36]. RGD patterns can be categorized into three types. A negative sign(λ2) indicates that [C_8_mim]BF_4_ and KRN molecules have strong interactions, such as hydrogen and halogen bonds. Sign(λ2) close to zero indicates weak interactions, such as the van der Waals force. A positive sign(λ2) indicates repulsion, such as steric hindrance. As shown in Figure 7A, multiple low-density peaks in the λ < 0 region indicate hydrogen or halogen bonding between [C_8_mim]BF_4_-KRN complexes. In the region where λ is close to 0, the green distribution peak indicates the presence of van der Waals forces between [C_8_mim]BF_4_ and KRN [37]. The electron-reduced density gradient region can be visualized in real molecular space using the RDG isosurface. As shown in Figure 7B, the anion of [C_8_mim]BF_4_ predominantly forms hydrogen bonds with KRN (blue), while the cation forms van der Waals forces with KRN (green).

Natural bond orbitals (NBO) were used to analyze the interactions between donors and receptors. Both anions and cations of ILs were found to have significant orbital overlap with KRN (Figure 7C). The second-order perturbation stabilization energy E(2) was used to assess the interaction degree in the [C_8_mim]BF_4_-KRN complex. A larger E(2) value indicates a stronger interaction between the electron donor and acceptor [38]. Notably, the E(2) value of the intermolecular orbitals formed by BF_4_^−^ and KRN was higher compared to that of the intermolecular orbitals formed by C_8_mim^+^ and KRN (Figure 7C). This suggests that BF_4_^−^ and KRN have more significant orbital overlap and reduce the system’s energy to a greater extent, contributing to the system’s stability [39]. Therefore, it is speculated that BF_4_^−^ plays a crucial role in the efficient extraction of PFS, which aligns with the experimental data of GIPF.

#### 2.6.3. MD Simulation

The [C_8_mim]BF_4_-KRN complex was used as a model to verify that a single [C_8_mim]BF_4_ can interact with KRN. Still, it may not comprehensively reflect the microscopic changes occurring across the entire system during the extraction of PFS. Therefore, this chapter simulates the forces of methanol and the [C_8_mim]BF_4_ system in the extraction process based on MD theory.

**Figure 8:**
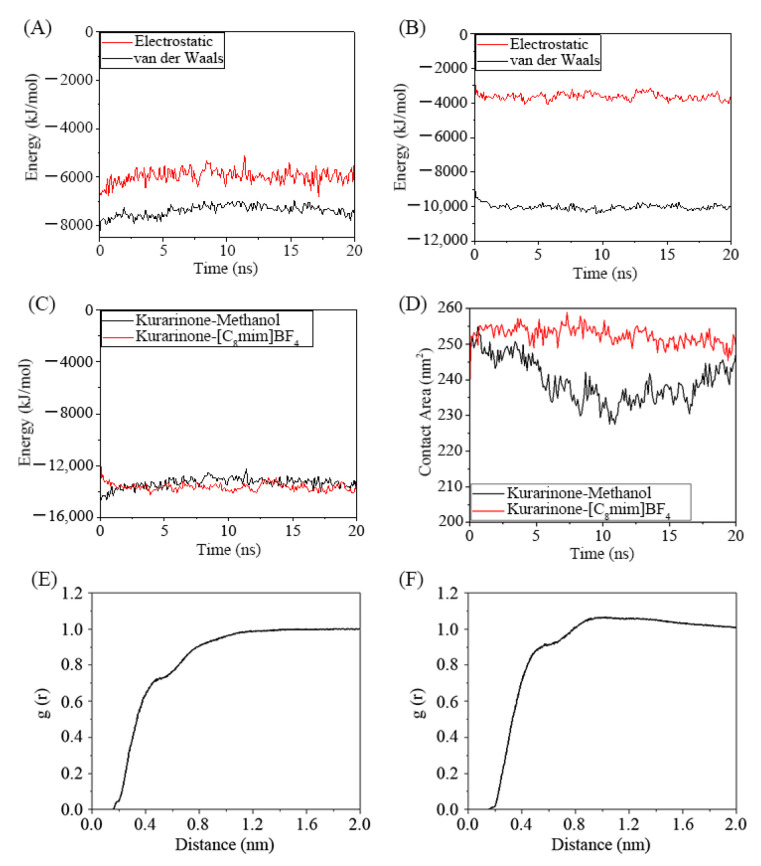
The interaction of KRN with the methanol and [C_8_mim]BF_4_ solvent system was simulated based on MD. (**A**) Interaction energy between KRN and methanol; (**B**) Interaction energy between KRN and [C_8_mim]BF_4_; (**C**) Comparison of the interaction energy of KRN with methanol and [C_8_mim]BF_4_; (**D**) Contact area of KRN with methanol and [C_8_mim]BF_4_ during simulation. The radial distribution function of methanol (**E**) and [C_8_mim]BF_4_ (**F**) around KRN.

In the simulation process, KRN was uniformly dispersed in the methanol and [C_8_mim]BF_4_ solvent without any agglomeration. During the extraction process, the methanol-KRN system’s interaction is mainly governed by van der Waals forces, with some electrostatic interactions present (Figure 8A). The van der Waals force in the [C_8_mim]BF_4_-KRN system is significantly greater than that in the methanol system. In contrast, the electrostatic interaction is weak (Figure 8B). Overall, the interaction force of the [C_8_mim]BF_4_-KRN system is greater than that of the methanol-KRN system. Additionally, the contact area between KRN and the two extraction solvents was assessed, revealing a significantly larger contact area between KRN and [C_8_mim]BF_4_ than between KRN and methanol. This suggests a stronger binding between KRN and [C_8_mim]BF_4_, consistent with the conclusion of a strong interaction between KRN and [C_8_mim]BF_4_.

To further examine the distribution of [C_8_mim]BF_4_ and methanol near KRN, we used the radial distribution function (RDF) to calculate the distribution information of the solvent. The RDF shows the likelihood of other particles being present around a specific particle [37]. The density of [C_8_mim]BF_4_ around KRN (Figure 8F) is higher than that of methanol molecules around KRN (Figure 8E). This indicates that [C_8_mim]BF_4_ has a stronger association with KRN, which is consistent with the interaction energy and contact area data for both systems.

In this section, the extraction mechanism of [C_8_mim]BF_4_ on PFS was systematically discussed for the first time from the perspective of surface morphology characterization and molecular interaction simulation. This analysis is helpful for improving our overall understanding of the extraction ability of ILs.

### 2.7. In Vitro Antitumor Activity of PFS

**Figure 9:**
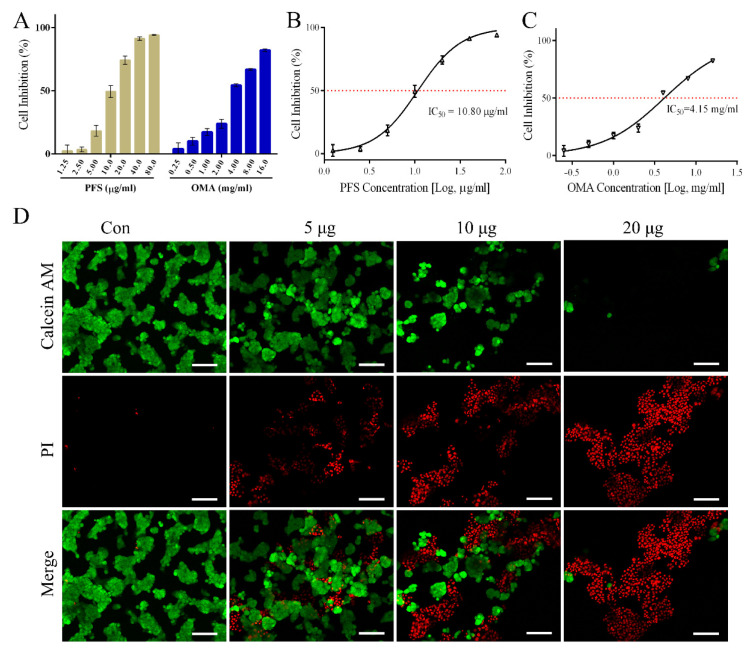
Effect of PFS on cytotoxicity in HepG2 cells. (**A**–**C**) Cell growth was determined by CCK8 assay; (**D**) Calcein−AM/PI staining was used to distinguish dead from living cells. Green fluorescence displays living cells and red fluorescence displays dead cells. Scale: 100 μm.

To evaluate the cytotoxic activity of PFS extracted based on ILs, oxymatrine (OMA), a widely recognized antitumor component of *S. flavescens*, was used as a control drug. Both PFS and OMA had cytotoxic effects (Figure 9A–C), and the IC_50_ value of PFS was 10.80 μg/mL, which was significantly lower than that of OMA (4.15 mg/mL). It was proved that the antitumor activity of PFS was stronger than that of alkaloids in *S. flavescens*. This was consistent with previous literature reports [1]. Furthermore, the results of AM-PI cell death and survival staining once again proved that PFS could inhibit HepG2 cell proliferation in a dose-dependent manner (Figure 9D). The anti-tumor mechanism of PFS is a complex process, which may be regulated by one or more signaling pathways, such as the regulation of the immune microenvironment [4], autophagy pathway [6], and apoptosis pathway [40]. The antitumor activity of PFS was strongly correlated with its structural properties [1]. Therefore, the anti-tumor mechanism and structure-activity relationship of PFS need to be further explored.

## 3. Materials and Methods

### 3.1. Materials and Chemicals

*S. flavescens* was purchased from Bei Jing Qian Cao Traditional Chinese Medicine Co., Ltd. (Beijing, China). HepG2 cells were purchased from the Chinese Academy of Sciences Typical Culture Preservation Committee cell bank (Shanghai, China). Kushenol I was purchased from Chengdu Chroma-Biotechnology Co. Ltd. (Chengdu, China). Kurarinone, sophoraflavanone G, 2′-methoxykurarinone, and isokurarinone were purchased from Baoji Earay Bio-Tech Co., Ltd. (Baoji, China). Matrine and oxymatrine were purchased from the National Institutes for Food and Drug Control (Beijing, China). Their purity is above 99%.

The Ionic liquids, including [C_8_mim]Br, [C_8_mim]PF_6_, [C_8_mim]HSO_4_, [C_8_mim]SbF_6_, [C_8_mim]CF_3_SO_3_, [C_8_mim]BF_4_, [C_2_mim]BF_4_, [C_4_mim]BF_4_, [C_6_mim]BF_4_, [C_10_mim]BF_4_, and [C_12_mim]BF_4_, were purchased from Shanghai Chengjie Chemical Reagent Co., LTD., (Shanghai, China), with a purity of >99%. Details of ILs are provided in Table S1. Deionized water was supplied by Watsons, and the acetonitrile and methanol were purchased from Fisher Scientific Worldwide Co., Ltd. (Shanghai, China). All other reagents are commercially available analytical-grade reagents.

### 3.2. Extraction of PFS Based on ILs

Branson 5510E-DTH (42 KHz) ultrasonic cleaner (Branson Ultrasonics Co., Ltd., (Shanghai, China) was used for ultrasonic-assisted extraction. The *S. flavescens* sample was initially crushed and filtered through a 50 mesh. Then, 0.5 g of the sample powder was placed in a 50 mL centrifuge tube, and 15 mL of ILs was added. After soaking for 8 hours, ultrasonic treatment was carried out at 60 °C for 30 min.

The mixture was centrifuged at room temperature at 8000 rpm for 5 min, then the supernatant was diluted with an equal amount of methanol, filtered with 0.22 μm membrane, and then quantitatively analyzed using HPLC (Supplementary Material). The extraction yield of the target compound was calculated according to Formula (2):

(2) Yieldmgg=weight of target compounds in extract mgweight of sample powder g

The weight of the sample powder represents the weight of the *S. flavescens* powder before extraction. The weight of the target compound in the extract can be obtained through HPLC analysis. All experiments and studies were conducted three times.

### 3.3. Optimization of Extraction Conditions

#### 3.3.1. Single-Factor Experimental Design

A series of single-factor experiments were conducted to optimize the ultrasonic-assisted extraction conditions for PFS. The default extraction conditions were as follows: solvent to solid ratio, 30 mL/g; extraction time, 40 min; soaking time, 8 h; extraction temperature, 60 °C. The other conditions remained the same when one of these factors was examined. The factors examined included the solvent-to-solid ratio (5, 10, 15, 20, 30, and 40 mL/g), extraction time (5, 10, 15, 20, 30, and 40 mL/g), soaking time (0, 2, 4, 6, 8, and 12 h), and extraction temperature (20, 30, 40, 60, 80, and 90 °C).

#### 3.3.2. Response Surface Design

Box-Bohnken design (BBD) was used to optimize the ultrasonic-assisted extraction conditions. According to the results of the single-factor experiment, a four-factor three-level experiment design was adopted (Table S5). The extraction yield of 5 prenylated flavonoids (KSI, SFG, KRN, MKR, IKR) with the highest content in *S. flavescens* were used as evaluation indexes to determine the optimal extraction conditions. The response was measured under the optimal extraction conditions. Finally, the experimental data were compared with the predicted value based on standard error, and the correctness of the model was verified.

### 3.4. Extraction of PFS Based on Conventional Solvents

The conventional solvent methanol and water were also used to extract *S. flavescens* powder by ultrasonic extraction, and compared with ILs. According to the previous research results of the research group, 0.5 g of sample powder was added to 13 mL of each extraction solvent, and ultrasonic extraction was carried out for 28 min at 20 °C. It was then centrifuged at room temperature at 8000 rpm for 5 min and the supernatant was taken. Before HPLC analysis, a 2-fold dilution with methanol and a 0.22 μm filter membrane was used.

Alkaloids are the most abundant compounds in *S. flavescens*. To verify the targeting of ILs extraction, we conducted a qualitative analysis of alkaloids and prenylated flavonoids in different extraction solutions. For the HPLC detection method of alkaloids, refer to Pharmacopoeia of the People’s Republic of China [41], and for the HPLC detection method of prenylated flavonoids, refer to Section 2.2.

### 3.5. PFS Recovery Based on Chromatographic Separation

We attempted to recover PFS from [C_8_mim]BF_4_ extract by chromatographic separation. ILs were separated, and PFS was recovered by Oasis HLB extraction cartridges (20 cc, 1 g; Waters, Milford, MA, USA) containing N-vinylpyrrolidone and divinylbenzene fillers. It was pretreated with 40 mL methanol and deionized water before column chromatography. [C_8_mim]BF_4_ extraction solution was added to the HLB column, and eluted with 100 mL (5 BV) 50% methanol and methanol in sequence. HPLC profiles of elution solutions at 210 and 295 nm were recorded, and it was observed that ILs were present in the 50% methanol eluent, while the PFS was present in the methanol eluent (Figure S3).

To characterize the chemical composition of the isolated PFS and evaluate its antitumor activity, PFS-rich freeze-dried powder (PFS-FP) was further prepared. Multiple column separations were carried out, and methanol eluent was combined, completely evaporated under vacuum rotation at 60 °C, re-suspended in an appropriate amount of water, and vacuum freeze-dried. PFS-FP was obtained.

### 3.6. Chemical Profile Analysis of PFS-FP Based on UHPLC-Q-Orbitrap MS

PFS-FP samples were completely dissolved with methanol, filtered through a 0.22 μm membrane before analysis on a Vanquish^TM^ Flex UHPLC instrument coupled with a Thermo Orbitrap Exploris 120 high-resolution mass spectrometer (Thermo Fisher Scientific, Waltham, MA, USA), and fitted with a heat electrospray ionization (HESI) interface. Samples were separated on an Acquity UPLC BEH C18 column (2.1 mm × 100 mm; 1.7 μm; Waters Corp., Milford, CT, USA). The column temperature was maintained at 35 °C. The flow rate was 0.20 mL/min. Mobile phase A was 0.1% (*v*/*v*) formic acid, and phase B was acetonitrile. The gradient program was as follows: 0–1 min, 0% B; 1–3 min, 0–5% B; 3–15 min, 5% B; 15–16 min, 5–8% B; 16–44 min, 8–90% B; 44–45 min, 90% B; 45–46 min, 90–0% B; 46–50 min, 0% B. The sample temperature was maintained at 4 °C, and the injection volume was 5 μL.

The operating conditions of the MS were as follows: HESI source in positive and negative mode; capillary temperature, 320 °C; sheath gas flow, 40 arb; auxiliary gas flow, 10 arb; scan modes, full MS and dd-MS^2^; full MS, 120,000 resolution; dd-MS^2^, 15,000 resolution; mass spectra recorded in *m*/*z* range, 100–1500; positive mode, 20 V/40 V/60 V HCD collision energy, and 3.5 kV spray voltage; negative mode, 10 V/20 V/30 V HCD collision energy, and 3.0 kV spray voltage. Data were analyzed with Xcalibur 3.0 (Thermo Fisher Scientific). The compounds were identified by chromatographic comparison against reference substances, laboratory databases, and literature reports [1,9].

### 3.7. Recyclability of ILs

To investigate the recyclability of [C_8_mim]BF_4_, the recovered [C_8_mim]BF_4_ was used to re-extract *S. flavescens* powder according to the experimental conditions described in Section 2.2. A total of 4 rounds of recovery and 5 rounds of ultrasonic extraction were conducted.

### 3.8. Fourier Transforms Infrared (FT-IR) Spectroscopy

FT-IR spectra (range 4000–400 cm^−1^) were recorded at 25 °C in a Frontier FT-IR spectrometer (PerkinElmer, Waltham, MA, USA).

### 3.9. Scanning Electron Microscopy (SEM)

In order to observe the morphological changes of *S. flavescens* powder before and after ultrasonic-assisted ILs extraction, Zeiss Sigma 300 SEM (Oberkochen, Germany) was used to capture images of *S. flavescens* powder at 3 kV acceleration voltage.

### 3.10. Quantum Chemical Calculations Based on Density Functional Theory (DFT)

Kurarinone (KRN) is one of the most abundant prenylated flavonoid compounds in *S. flavescens.* The interaction between ILs and PFS during extraction was simulated based on DFT to explore the interaction between [C_8_mim]BF_4_-KRN molecules. The geometries of MeOH, KRN, [C_8_mim]BF_4_, and the complex of kurarinone+[C_8_mim]BF_4_ were all optimized with dispersion corrected density functional theory (DFT-D3) at the B3LYP-D3/6-31+G(d) level [42]. All these DFT calculations were performed using the Gaussian 16 program suite (Gaussian, Inc., Wallingford, CT, USA, 2016). The visualization of the ESP mapping was all rendered using Gauss View. The Natural Bond Orbital 3.1 program integrated with the Gaussian program suite package was used to obtain the NBO and natural population of the optimized structures. The visualization of the NBO orbitals was all rendered using the Visual Molecular Dynamic program. It is difficult to obtain the interaction energy of each hydrogen bond because every molecule has more than two hydrogen bonds with other molecules simultaneously. Therefore, we predict the bond strength using a descriptor of electron density at bond critical points (BCPs) [35], which can treat intramolecular hydrogen bonds. The bond critical point of these three hydrogen bonds was searched using the Multiwfn program. The non-covalent interaction analysis, which was also named reduced density gradient (RDG) analysis, was also performed using the Multiwfn program to study the weak intermolecular interaction visually.

### 3.11. Molecular Dynamics (MD) Simulation

In order to explore the interaction force inside the solution system during the extraction process, the following MD simulation process was carried out based on the KRN, methanol, and [C_8_mim]BF_4_ models calculated by quantum chemical DFT. The atomic charge distribution was derived from the above DFT calculation, and other bonds and non-bond interaction parameters were obtained by sobtop. All-atom MD simulations were conducted using the GROMACS software package, version 2021.5. The AMBER03 force field was employed to describe the molecule. Fifty KRN molecules were randomly placed in a 10 nm × 10 nm × 10 nm cube box, and 2000 [C_8_mim]BF_4_ ion pairs were added into the [C_8_mim]BF_4_ system. In the methanol system, 14130 methanol molecules were added. Before the MD simulation, the steepest descent method was used to minimize the energy of the two systems. A short simulation of 500 ps was then performed using the NVT and NPT systems, respectively. In this process, position restrictions are imposed on the heavy atoms to dissolve the molecules within the system completely. Subsequently, the location restrictions were lifted, and the NPT system was used for data production operations lasting 20 ns. The pressure was maintained at *p* = 1.0 bar using a Parrinello-Rahman barometer, and the temperature was controlled at 298.15 K using a velocity scale thermostat with a coupling constant τ = 0.1 ps. The non-covalent interaction was calculated with 1.2 nm as the cutoff point, and the remote electrostatic interaction was calculated with the particle grid Ewald summation method. The LINCS algorithm was used to constrain all hydrogen bonds. The simulation time step is 2 fs, and the neighbor list is updated every 10 steps. Periodic boundary conditions are applied in all three directions. Visualization was performed using PyMOL-3.0.3.

### 3.12. Antitumor Activity of PFS In Vitro

#### 3.12.1. Cell Culture

HepG2 cells medium conditions: 95% DMEM high glucose medium (Solarbio Technology Co., LTD., Beijing, China) + 5% FBS (Sigma, Livonia, MI, USA). Cells were cultured in a humid, closed cell incubator at 37 °C, 5% CO_2_. After digestion with 0.1% pancreatin for 1 min, the cells were collected, dispersed, and cultured at a ratio of 1:3. The experiment used logarithmic growth phase cells.

#### 3.12.2. Cell Cytotoxic Assays

8 × 10^3^. HepG2 cells per well were seeded in 96-well plates and incubated overnight. The cells were incubated with 100 μL of drug-containing medium at various concentrations (PFS, 1.25–80 μg/mL; OMA, 0.25–16 mg/mL) for 24 h. Then, 10 μL of CCK-8 solution was added to each well and incubated for an additional hour. OD values at 450 nm were measured by microplate spectrophotometer, and cell viability was calculated.

#### 3.12.3. Calcein-AM/PI Double Staining

1 × 10^5^. HepG2 cells per well were seeded in a 35 mm glass bottom confocal culture dish and incubated overnight. The cells were treated with the drug-containing medium (5, 10, and 20 μg/mL) for 24 h. An AM/PI working solution was used to incubate the cells at 37 °C without light for 30 min according to the kit instructions (Beyotime Biotechnology, Shanghai, China). A Leica (DMI 600B, Wetzlar, Germany) confocal microscope was used to record 4 random images in each group. Green fluorescence’s excitation and emission wavelength is 494/517 nm, and red fluorescence’s excitation and emission wavelength is 535/617 nm.

### 3.13. Statistical Analysis

SPSS 25 statistical software (IBM; Armonk, NY, USA) was used for statistical analysis. All experimental data are expressed as mean ± standard deviation. Statistical differences between groups were evaluated by one-way analysis of variance (ANOVA). *p* < 0.05 was considered statistically significant. GraphPad Prism 6.0.2 software was used for image processing.

## 4. Conclusions

This study used ultrasound-assisted ILs to efficiently and selectively extract prenylated flavonoids from *S. flavescens*. Among all the hydrophilic and hydrophobic ILs, [C_8_mim]BF_4_ showed the best extraction effect on five representative prenylated flavonoids (KSI, KRN, SFG, MKR, IKR). Under the optimal extraction conditions, the yield of PFS was 7.38 mg/g. [C_8_mim]BF_4_ extraction efficiency and selectivity were much higher than that of the conventional reagent methanol. The active part of prenylated flavonoids was successfully recovered from ILs using reversed-phase solid-phase extraction. In total, 39 kinds of prenylated flavonoids were identified in PFS-FP based on UHPLC-Q-Orbitrap MS. The exploration of the extraction mechanism showed that [C_8_mim]BF_4_ could destroy the microstructure of the cell wall of *S. flavescens*. The strong interaction in the [C_8_mim]BF_4_-KRN solution system was revealed. In addition, the potent antitumor activity of PFS was demonstrated based on HepG2 cell experiments. In general, this study provides a possible method for the targeted extraction of strong pharmacological active ingredients in herbal medicine. It offers another analytical approach for the in-depth analysis of the extraction mechanism of active ingredients.

## Figures and Tables

**Table 1:** Box-Behnken response surface test design and results.

Std	Run	A: Solvent to Solid Ratio (mL/g)	B: Extraction Time (min)	C: Soaking Time (h)	D: Extraction Temperature (°C)	Y: Total Flavonoid (mg/g)
1	6	20	30	6	60	7.04
2	20	40	30	6	60	6.94
3	12	20	50	6	60	6.88
4	27	40	50	6	60	6.74
5	26	30	40	4	40	6.98
6	17	30	40	8	40	7.06
7	19	30	40	4	80	6.94
8	21	30	40	8	80	7.09
9	7	20	40	6	40	7.21
10	2	40	40	6	40	6.74
11	10	20	40	6	80	6.89
12	8	40	40	6	80	6.97
13	3	30	30	4	60	6.97
14	22	30	50	4	60	6.97
15	5	30	30	8	60	7.09
16	13	30	50	8	60	6.79
17	9	20	40	4	60	7.03
18	25	40	40	4	60	6.78
19	23	20	40	8	60	7.14
20	14	40	40	8	60	6.74
21	11	30	30	6	40	7.00
22	1	30	50	6	40	6.94
23	16	30	30	6	80	6.97
24	24	30	50	6	80	6.71
25	18	30	40	6	60	7.35
26	29	30	40	6	60	7.25
27	4	30	40	6	60	7.37
28	28	30	40	6	60	7.32
29	15	30	40	6	60	7.33

**Table 2:** ANOVA for response surface quadratic model.

Source	Sum of Squares	df	Mean Square	F-Value	*p*-Value	
Model	154.16	14	11.01	15.03	<0.0001	significant
A	12.14	1	12.14	16.57	0.0011	significant
B	13.4	1	13.4	18.28	0.0008	significant
C	5.08	1	5.08	6.94	0.0196	significant
D	1.86	1	1.86	2.53	0.1338	
AB	0.0042	1	0.0042	0.0058	0.9405	
AC	1.59	1	1.59	2.17	0.1632	
AD	14.06	1	14.06	19.19	0.0006	significant
BC	4.08	1	4.08	5.57	0.0333	significant
BD	1.84	1	1.84	2.51	0.1358	
CD	0.245	1	0.245	0.3344	0.5723	
A^2^	36.73	1	36.73	50.12	<0.0001	significant
B^2^	60.28	1	60.28	82.26	<0.0001	significant
C^2^	17.44	1	17.44	23.8	0.0002	significant
D^2^	36.08	1	36.08	49.23	<0.0001	significant
Residual	10.26	14	0.7328			
Lack of Fit	7.76	10	0.7757	1.24	0.4515	not significant
Pure Error	2.5	4	0.6255			
Cor Total	164.42	28				

**Table 3:** GIPF parameter values based on ESP distribution.

	σ_+_^2^ (kcal/mol)^2^	σ_−_^2^ (kcal/mol)^2^	σ_tot_^2^ (kcal/mol)^2^
MeOH	90.41	137.45	227.86
Kurarinone	180.33	88.46	268.78
C8mim-BF4	151.10	360.45	511.55

**Table 4:** Topological parameters at BCPs for Kurarinone/[C_8_mim]BF_4_.

H-Bond	*ρ*_BCP_ (a.u).	*V*^2^*ρ*_BCP_ (a.u)	*V*_CP_ (a.u)	*η*_BCP_ (a.u)	λ1	λ2	λ3	E_HB_ (kcal/mol)
F_103_… H_72_	0.0133	0.0503	−0.0112	0.1961	−0.0160	−0.0155	0.0820	−2.2417
F_101_… H_60_	0.0141	0.0532	−0.0123	0.1964	−0.0168	−0.0158	0.0859	−2.4179
F_101_… H_41_	0.0135	0.0536	−0.0117	0.1804	−0.0150	−0.0148	0.0835	−2.2864
F_102_… H_49_	0.0411	0.1350	−0.0403	0.2582	−0.0713	−0.0698	0.2761	−8.4351
O_4_… H_77_	0.0151	0.0469	−0.0114	0.2114	−0.0168	−0.0158	0.0796	−2.6466
O_1_… H_89_	0.0121	0.0394	−0.0088	0.1982	−0.0128	−0.0124	0.0647	−1.9629
F_104_… H_68_	0.0285	0.0960	−0.0277	0.2320	−0.0409	−0.0396	0.1766	−5.6349

## Data Availability

All data and materials are present in the manuscript and Supplementary Materials.

## Supplementary Materials

The following supporting information can be downloaded at: https://www.mdpi.com/article/10.3390/molecules30030500/s1, Figure S1: The contour map of the influence of different factors on the composite valuation. Figure S2: Extraction yield of KSI, KRN, SFG, MKR, and IKR from *S. flavescens* by [C8mim]BF4 and methanol. Figure S3: HPLC chromatogram of 50% methanol eluent and methanol eluent. Left, chromatogram at 295 nm; right, chromatogram at 210 nm. KSI, kushenol I; KRN, kurarinone; SFG, sophoraflavanone G; MKR, 2’-methoxykurarinone; IKR, isokurarinone. Figure S4: Recyclability of [C8mim]BF4 for extracting prenylated flavonoids from *S. flavescens*. R-0, extraction using the original [C8mim]BF4; R-1, extraction using [C8mim]BF4 recovered once; R-2, extraction using [C8mim]BF4 recovered twice; R-3, extract using [C8mim]BF4 that has been recovered three times; R-4, extraction using [C8mim]BF4 recovered four times; **** Most significant (*p* < 0.001). Figure S5: ESP maps and area percent in each range on the vdW surface. Figure S6: Representative snapshots of the initial (0 ns) and final (20 ns) moments of kurarinone in methanol and [C8mim]BF4 solvents.; Table S1: Names and structures of ionic liquids. Table S2: Calibration curves, linearity, precision, stability and repeatability of five compounds analysis with HPLC. Table S3: Standard recovery of five compounds analysis with HPLC. Table S4: Recovery rates of the five analytes. Table S5: Factors and levels of the Box-Bokhen design. Table S6: ANOVA for response surface quadratic model. Table S7: MS data of compounds in PFS freeze-dried powder determined by UHPLC-Q-Orbitrap. Table S8: The energies of kurarinone/[C8mim]BF4 system, kurarinone, and [C8mim]BF4.
