# Supplementary material for: Efficient and Selective Extraction of Prenylated Flavonoids from Sophora flavescens Using Ultrasound-Assisted Hydrophobic Ionic Liquid and Characterization of Extraction Mechanism
Source: Molecules. 2025 Jan 23;30(3):500. doi: 10.3390/molecules30030500 (PMC11820926; doi:10.3390/molecules30030500)
Supplement: Supplementary file 1 [file molecules-30-00500-s001.zip › molecules-3394267-supplementary.pdf]

Efficient and Selective Extraction of Prenylated Flavonoids from *Sophora flavescens* Using Ultrasound-Assisted Hydrophobic Ionic Liquid and Characterization of Extraction Mechanism

[1]

Shasha Kong [1,†], Qian Liao [1,†], Yuling Liu [1], Ruying Tang[1], Longfei Lin[1,*], Hui Li[1,2,*]

[1] Institute of Chinese Materia Medica, China Academy of Chinese Medical Sciences, Nanxiaojie 16, Dongzhimennei Ave, 100700 Beijing, P.R. China

[2] Institute of Traditional Chinese Medicine Health Industry, China Academy of Chinese Medical Sciences, 330006, Jiangxi, P.R. China

[†] These authors contributed equally to this work

***Correspondence author**

E-mail: lflin@icmm.ac.cn (L. Lin), hli1967@icmm.ac (H. Li)

[1]**Abbreviations**: AIM, atoms in molecules; BCP, bond critical point; ESP, electrostatic surface potential; DFT, density functional theory; FT-IR, fourier infrared spectroscopy; GIPF, general interaction properties function; HPLC, high performance liquid chromatograph; IKR, isokurarinone; ILs, ionic liquids; KRN, kurarinone; KSI, kushenol I; MD, molecular dynamics; MKR, 2'-methoxykurarinone; MPa, mega pascal; NBO, Natural bond orbitals; PFS, prenylated flavonoids in *Sophora flavescens*; PFS-FP, PFS-rich freeze-dried powder; RDG, reduced density gradient; RP-SPE, reversed-phase solid-phase extraction; SEM, Scanning electron microscopy; SFG, sophoraflavanone G; TCM, traditional Chinese medicine; UA-$[C_8mim]BF_4$, ultrasound assisted $[C_8mim]BF_4$

S1. HPLC Method for the Determination of PFS and $[C_8mim]BF_4$

S1.1 HPLC Method

Both determination of PFS and $[C_8mim]BF_4$ were performed on a Waters 2695 HPLC instrument (Mass, USA). Samples were separated on an Inertsil® ODS-HL column (250 mm × 4.6 mm, 5 μm; Shimadzu, Kyoto, Japan). The column temperature was maintained at 30 °C. The flow rate is 0.80 ml/min. Mobile phase A consisted of a 0.1% (v/v) aqueous formic acid solution, while mobile phase B comprised methanol. The elution procedure was as follows: 0-5 min, 50% B; 5-25 min, 50-70% B; 25-35 min, 70% B; 35-50 min, 70-90% B; 50-59 min, 90% B; 59-60 min, 90-50% B; 60-66 min, 50% B. The sample temperature was maintained at 20 °C, and the injection volume was 10 μL. The five prenylated flavonoids were recorded at 254 nm, and $[C_8mim]BF_4$ was recorded at 254 nm. KSI, SFG, KRN, MKR, and IKR in the chromatograms could be recognized by comparing them with the corresponding standards.

S1.2. Method Validation of HPLC Analysis

S1.2.1 Linearity

The linearity of the quantitative method was evaluated in the KRN concentration range of 14.40 to 460 μg/ml; the calibration curve is $y = 2.321\times10^7\ x + 61788.664$ (R^2=1.0000). The linearity of the quantitative method was evaluated in the SFG concentration range of 7.50 to 240 μg/mL; the calibration curve is $y = 3.594\times10^7\ x + 27428.716$ (R^2=1.0000). The linearity of the quantitative method was evaluated in the KSI concentration range of 7.50 to 240 μg/mL; the calibration curve is $y = 2.773\times10^7\ x + 49310.259$ (R^2=0.9998). The linearity of the quantitative method was evaluated in the MKR concentration range of 1.25 to 80 μg/mL; the calibration curve is $y = 1.521\times10^7\ x + 3658.453$ (R^2=0.9998). The linearity of the quantitative method was evaluated in the IKS concentration range of 1.25 to 80 μg/mL; the calibration curve is

$y = 1.755\times10^7 x + 4774.791$ (R^2=0.9998, Table S2).

S1.2.2 Precision

The method's precision was evaluated by determining the five compounds in one sample in six replicates daily and duplicating the experiments on three consecutive days. The intra- and interday precision was represented by relative standard deviations (RSD). KRN, SFG, KSI, MKR, and IKS showed good intra- and inter-day precision. The RSD values of intra-day precision were 0.475, 0.538, 0.485, 0.461, and 0.756; The RSD values of inter-day precision were 0.522, 0.764, 0.541, 1.046, and 0.887, respectively (Table S2).

S1.2.3 Stability

The stability of the method was evaluated by analyzing sample after being exposed in the autosampler at 4 °C for 0, 2, 4, 6, 8, 12, 24, and 48 h, respectively. The RSD of KRN, SFG, KSI, MKR, and IKS are 1.026 %, 1.074, 1.108, 1.080 and 1.192 %, respectively (Table S2).

S1.2.4 Repeatability

The repeatability of the method was evaluated by detecting five compounds in six sample solutions prepared in parallel. The RSD of KRN, SFG, KSI, MKR, and IKS are 1.774 %, 1.792, 1.819, 1.746, and 1.624 %, respectively (Table S2). This indicates that the method has good repeatability.

S1.2.5 Recovery

Standard KRN, SFG, KSI, MKR, and IKS solutions were added to the known extract concentrations to achieve spiked treatment. The KRN recoveries ranged from 98.98 to101.37%, SFG recoveries ranged from 97.77 to 100.66%, KSI recoveries ranged from 98.01 to 100.82%, MKR recoveries ranged from 96.73 to 101.60%, and IKS recoveries

ranged from 97.67 to 100.95%. The RSD values of five compounds were less than 2% (Table S3).

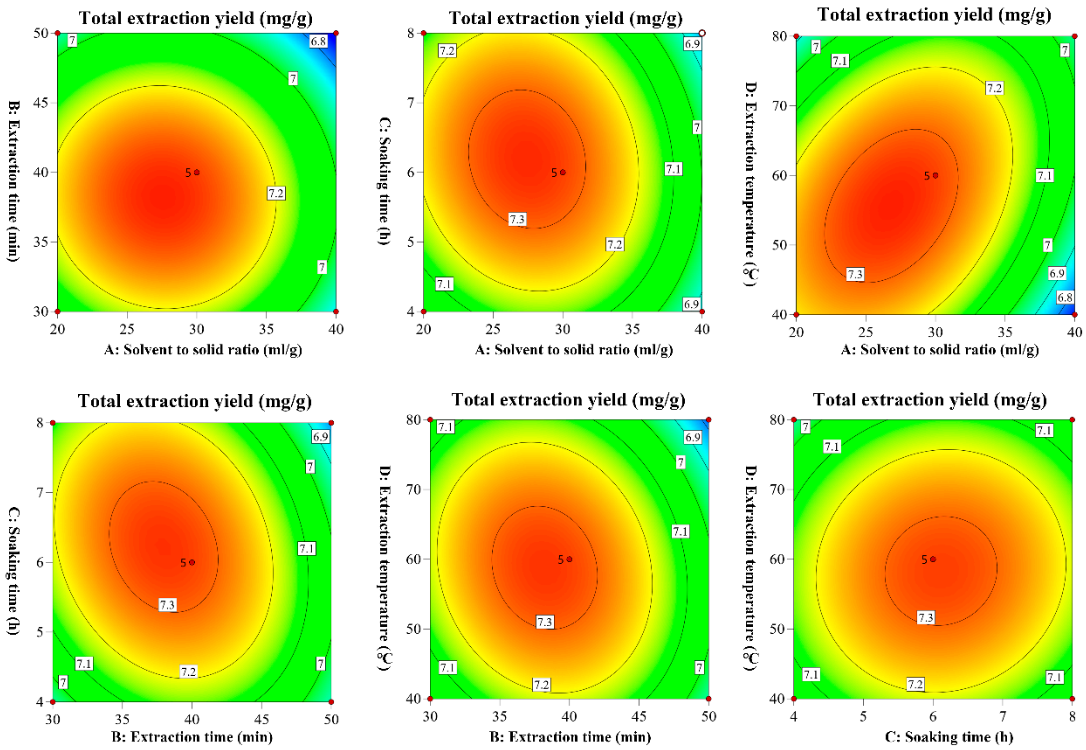

Figure S1. The contour map of the influence of different factors on the composite valuation.

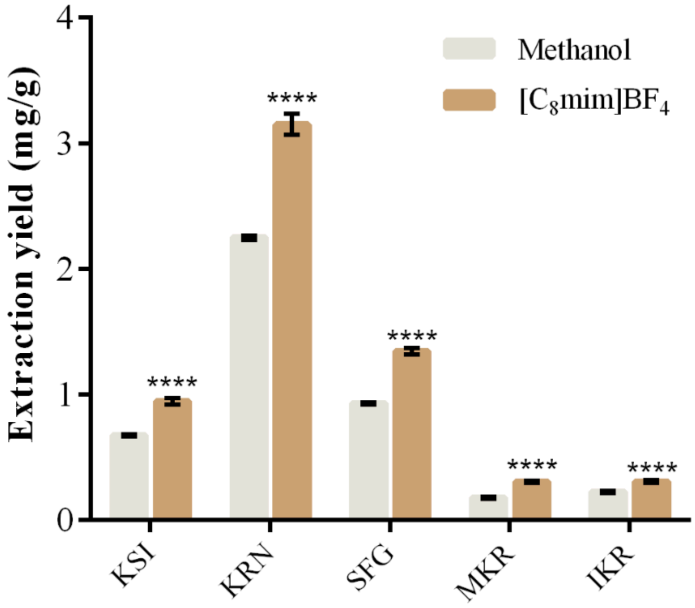

Figure S2. Extraction yield of KSI, KRN, SFG, MKR, and IKR from *S. flavescens* by [C_8mim]BF_4 and methanol.

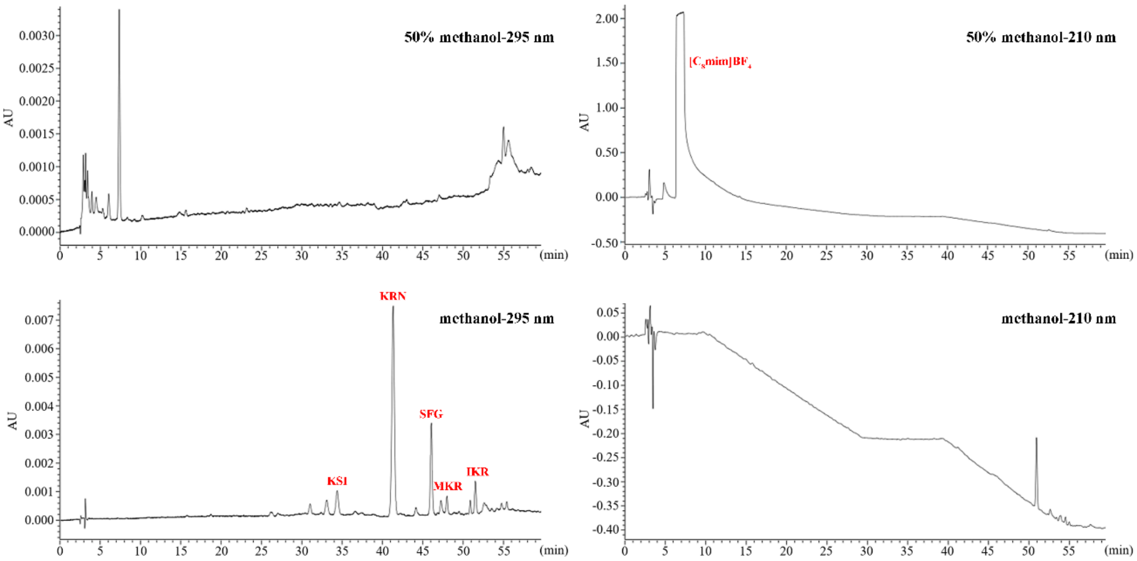

Figure S3. HPLC chromatogram of 50% methanol eluent and methanol eluent. Left, chromatogram at 295 nm; right, chromatogram at 210 nm. KSI, kushenol I; KRN, kurarinone; SFG, sophoraflavanone G; MKR, 2'-methoxykurarinone; IKR, isokurarinone.

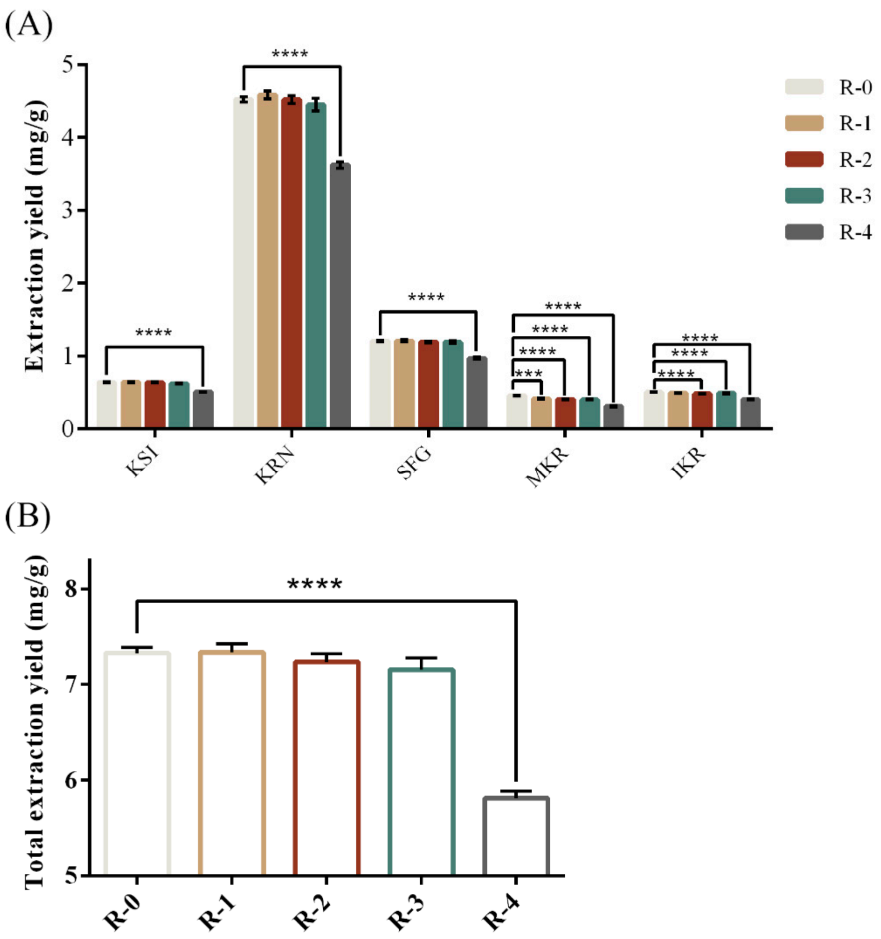

Figure S4. Recyclability of [C_8mim]BF_4 for extracting prenylated flavonoids from *S. flavescens*. R-0, extraction using the original [C_8mim]BF_4; R-1, extraction using [C_8mim]BF_4 recovered once; R-2, extraction using [C_8mim]BF_4 recovered twice; R-3, extract using [C_8mim]BF_4 that has been recovered three times; R-4, extraction using [C_8mim]BF_4 recovered four times; **** Most significant ($p < 0.001$).

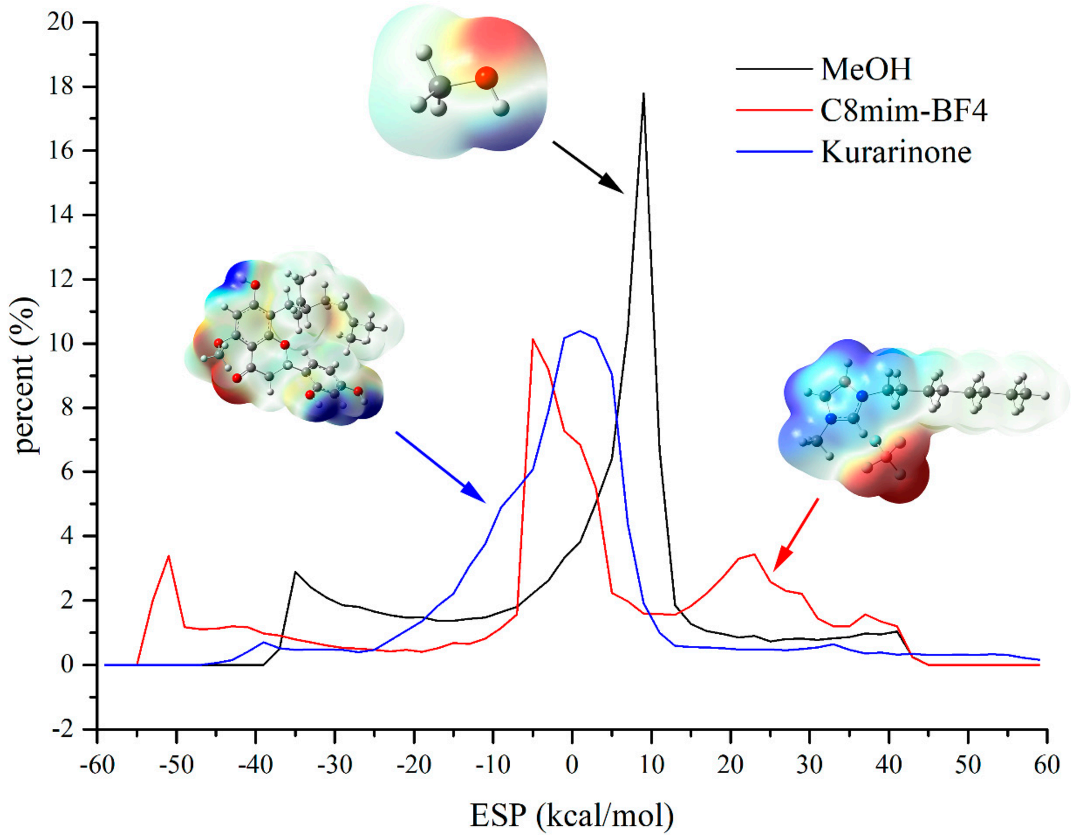

Figure S5. ESP maps and area percent in each range on the vdW surface.

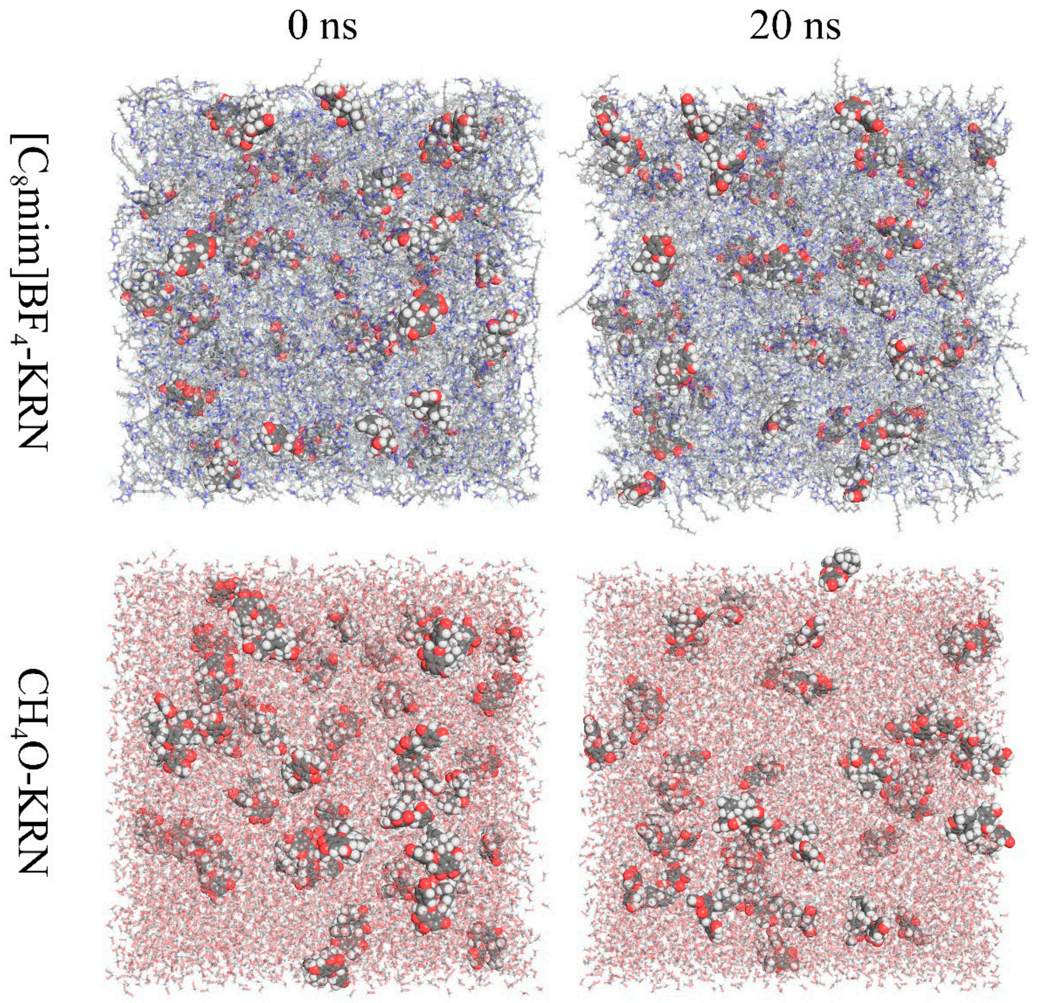

Figure S6. Representative snapshots of the initial (0 ns) and final (20 ns) moments of kurarinone in methanol and $[C_8mim]BF_4$ solvents.

Table S1. Names and structures of ionic liquids.

Cations	Anions	Names (Abbreviations)
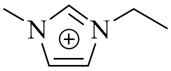	BF_4^-	$[C_2mim]BF_4$
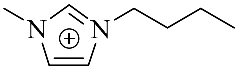	BF_4^-	$[C_4mim]BF_4$
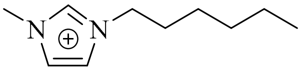	BF_4^-	$[C_6mim]BF_4$
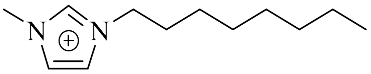	BF_4^-	$[C_8mim]BF_4$
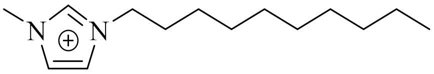	BF_4^-	$[C_{10}mim]BF_4$
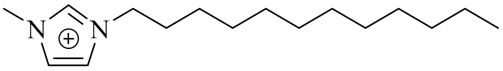	BF_4^-	$[C_{12}mim]BF_4$
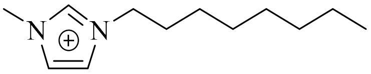	Br^-	$[C_8mim]Br$
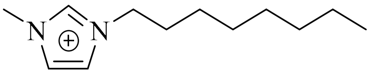	HSO_4^-	$[C_8mim]HSO_4$
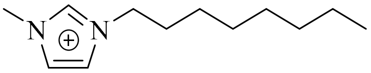	PF_6^-	$[C_8mim]PF_6$
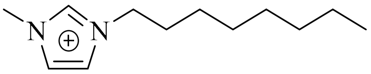	SbF_6^-	$[C_8mim]SbF_6$
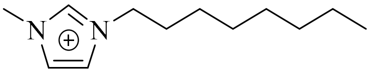	$CF_3SO_3^-$	$[C_8mim]CF_3SO_3$

Table S2. Calibration curves, linearity, precision, stability and repeatability of five compounds analysis with HPLC

Compound	Calibration Curve	R^2	Linear Range (μg/mL)	Intra-day Precision RSD (%)	Inter-day Precision RSD (%)	Stability RSD (%)	Repeatability RSD (%)
KRN	$y = 2.321\times10^7 x + 61788.664$	1.0000	14.40-460	0.475	0.522	1.026	1.774
SFG	$y = 3.594\times10^7 x + 27428.716$	1.0000	7.50-240	0.538	0.764	1.074	1.792
KSI	$y = 2.773\times10^7 x + 49310.259$	0.9998	7.50-240	0.485	0.541	1.108	1.819
MKR	$y = 1.521\times10^7 x + 3658.453$	0.9998	1.25-80	0.461	1.046	1.080	1.746
IKS	$y = 1.755\times10^7 x + 4774.791$	0.9998	1.25-80	0.756	0.887	1.192	1.624

Table S3. Standard recovery of five compounds analysis with HPLC

Compound	Original Content (μg)	Spiked Content (μg)	Detected Content (μg)	Recovery (%)	RSD (%)
KRN	642.00	682.95	1317.98	98.98	0.90
	644.89	682.95	1322.01	99.15	
	715.76	682.95	1393.24	99.20	
	767.71	682.95	1451.72	100.16	
	651.94	682.95	1333.61	99.81	
	663.49	682.95	1355.80	101.37	
SFG	281.82	298.20	578.22	99.40	1.16
	298.61	298.20	598.78	100.66	
	304.58	298.20	603.39	100.21	
	303.05	298.20	597.06	98.59	
	277.52	298.20	569.07	97.77	
	281.27	298.20	574.01	98.17	
KSI	142.00	149.10	288.14	98.01	1.07
	143.49	149.10	293.81	100.82	
	152.15	149.10	300.59	99.56	
	155.77	149.10	303.71	99.22	
	142.92	149.10	289.46	98.28	
	143.78	149.10	293.02	100.10	
MKR	46.05	41.14	86.78	99.02	1.70
	48.14	41.14	88.53	98.17	
	51.43	41.14	92.60	100.07	
	50.81	41.14	91.93	99.95	
	44.78	41.14	84.57	96.73	
	48.74	41.14	90.54	101.60	
IKS	54.32	68.88	122.56	99.06	1.28
	59.08	68.88	128.61	100.95	
	57.83	68.88	125.41	98.11	
	63.56	68.88	132.53	100.14	
	55.76	68.88	123.50	98.36	
	53.54	68.88	120.81	97.67	

Table S4. Recovery rates of the five analytes

Analyte	Solid phase extraction (%)
KSI	75.12 ± 2.06
KRN	62.32 ± 0.99
SFG	85.15 ± 0.63
MKR	84.49 ± 1.62
IKR	82.15 ± 1.33

Table S5. Factors and levels of the Box-Bokhen design

Factor	Units	Level		
		-1	0	1
A: Solvent to solid ratio	ml/g	20	30	40
B: Extraction time	min	30	40	50
C: Soaking time	h	4	6	8
D: Extraction temperature	℃	40	60	80

Table S6. ANOVA for response surface quadratic model

Variable	Sum of squares
R^2	0.9444
Adjusted R^2	0.8888
Predicted R^2	0.7136
Adep Precision	13.9378
C.V.%	0.9180

Table S7. MS data of compounds in PFS freeze-dried powder determined by UHPLC-Q-Orbitrap

NO.	T(min)	Formular	$[M-H]^-$ Predicted	Measured	Error (ppm)	Fragment ion	Compound
**1**	27.3	$C_{26}H_{32}O_8$	471.201	471.202	1.02	471.20(100), 177.01(63.33), 293.17(46.87), 149.02(13.21)	Kushenol K
**2**	28.0	$C_{26}H_{32}O_7$	455.206	455.207	0.94	455.20(41.26), 161.02(100), 293.17(57.85)	Kurarinol
**3**	28.5	$C_{26}H_{32}O_7$	455.206	455.208	0.75	161.02(100), 293.17(50.65), 455.21(40.60)	(2R,3R)-5-Methoxy-7,4'-dihydroxy-8-[3,3-dimethylallyl]-flavanonol
**4**	28.6	$C_{21}H_{22}O_6$	369.133	369.134	1.05	369.13(17.90), 161.02(100), 207.10(48.19), 135.04(6.69)	5-Methoxy-7,2',4'-trihydroxy-8-[3,3-dimethylallyl]-flavanone
**5**	29.1	$C_{21}H_{22}O_6$	369.133	369.134	0.93	369.13(100), 207.10(39.05), 341.13(24.95), 325.14(5.56), 192.0788(2.72)	Sophoraflavanone M
**6**	30.0	$C_{25}H_{30}O_7$	441.191	441.192	0.99	441.19(100), 279.16(86.07), 161.02(34.01), 211.11(1.30), 305.13(1.52), 331.15(1.24), 423.17(0.65)	Norkurarinol/isomeride
**7**	30.1	$C_{21}H_{22}O_5$	353.138	353.139	0.81	353.13(100), 119.05(40.26), 233.08(28.44), 165.09(3.25), 218.05(2.22)	Isoxanthohumol
**8**	30.2	$C_{20}H_{20}O_6$	355.118	355.119	1.04	355.11(59.21), 327.12(27.54), 193.08(17.97)	Kievitone
**9**	30.6	$C_{20}H_{20}O_6$	355.118	355.119	1.01	355.11(59.21), 193.08(100), 161.02(37.33)	Leachianone G
**10**	30.7	$C_{21}H_{22}O_7$	385.128	385.129	1.00	385.12(100), 193.08(88.97), 191.03(29.96), 357.13(6.60)	Kushenol V/Kushenol W
**11**	31.0	$C_{25}H_{30}O_7$	441.191	441.192	0.99	441.19(100), 279.16(84.09), 161.02(33.03), 211.17(3.74), 305.13(2.02), 331.15(1.04), 237.14(1.03), 423.18(0.58)	Norkurarinol
**12**	31.2	$C_{20}H_{20}O_4$	323.128	323.129	0.93	323.12(100), 203.07(20.30), 119.05(33.26), 135.24(9.65)	Isobavachin
**13**	31.5	$C_{26}H_{30}O_7$	453.191	453.191	0.53	453.19(100), 177.01(77.73), 275.16(42.98), 149.02(17.60), 435.18(2.64)	Kushenol I*
**14**	32.0	$C_{26}H_{32}O_7$	455.206	455.208	1.06	455.20(100), 305.13(10.71), 149.06(5.15), 331.15(3.23), 261.14(2.16)	Kushenol Q/Kushenol P
**15**	32.2	$C_{26}H_{30}O_6$	437.196	437.196	0.40	437.19(24.20), 161.02(100), 275.16(42.43), 151.03(3.85), 419.18(1.47)	Kurarinone*
**16**	32.3	$C_{26}H_{28}O_7$	451.175	451.176	0.93	451.17(100), 301.14(8.12), 149.02(5.06)	5-methylkushenol C
**17**	32.4	$C_{20}H_{24}O_4$	327.159	327.160	0.91	327.16(100), 165.05(16.88), 133.06(11.63), 149.09(3.40)	5-methoxy-7-hydroxy-8-lavandulylbenzochromone

**18**	32.6	$C_{25}H_{28}O_7$	439.175	439.176	0.90	439.17(100), 261.14(47.35), 177.01(16.16), 149.02(3.83), 411.18(2.63), 193.15(2.14)	Kushenol L
**19**	32.6	$C_{20}H_{18}O_6$	353.102	353.103	0.98	353.10(100), 298.04(8.12), 219.06(2.45), 164.01(0.82), 325.10(1.05)	Noranhyioicaritin/8-prenylkaempferol
**20**	32.8	$C_{21}H_{22}O_6$	369.133	369.134	1.02	369.13(100), 219.06(15.06), 149.06(7.02)	Kenusanone I
**21**	33.0	$C_{27}H_{32}O_7$	467.206	467.208	1.09	467.20(100), 275.16(17.01), 439.21(15.31), 150.03(3.12)	2'-methoxy kushenol I
**22**	33.1	$C_{26}H_{30}O_6$	437.196	437.197	1.04	437.19(100), 409.20(16.39), 275.16(27.21), 161.02(2.66)	Kosamol R
**23**	33.2	$C_{26}H_{32}O_7$	455.206	455.208	1.15	455.20(100), 305.13(14.62), 331.15(3.09), 149.06(6.95), 123.04(4.59)	Kuraridinol
**24**	33.5	$C_{25}H_{26}O_6$	421.165	421.166	1.09	421.16(100), 119.05(28.25), 301.14(23.51)	6,8-diprenylkaempferol
**25**	33.7	$C_{26}H_{30}O_5$	421.201	421.202	1.05	421.20(100), 119.05(28.25), 301.14(23.51), 233.15(2.85), 163.00(4.60)	Kushenol U/Kushenol R
**26**	33.7	$C_{25}H_{26}O_7$	437.160	437.161	1.01	437.16(100), 314.04(3.56), 299.12(0.99), 287.12(2.69)	Kushenol C
**27**	34.0	$C_{27}H_{32}O_6$	451.212	451.212	0.86	451.21(100), 149.06(26.20), 301.14(20.99), 163.00(3.92), 134.03(2.74)	2'-methoxykurarinone*
**28**	34.1	$C_{20}H_{20}O_4$	323.128	323.129	1.09	323.10(50.07), 265.14(100), 68.05(6.08)	Glabranin
**29**	34.2	$C_{25}H_{28}O_6$	423.180	423.181	0.61	423.18(78.92), 261,14(100), 161.02(48.09), 137.02(5.42), 193.15(3.31), 287.12(1.84)	Sophoraflavanone G*
**30**	34.9	$C_{25}H_{28}O_7$	439.175	439.176	0.80	439.17(100), 261.14(42.62), 177.01(17.29), 149.02(5.11), 192.07(5.53), 421.16(3.70)	Kushenol X
**31**	36.1	$C_{25}H_{28}O_5$	407.185	407.186	0.98	407.18(100), 287.12(9.93), 119.05(5.75), 164.01(4.69)	Kushenol A
**32**	36.5	$C_{26}H_{30}O_6$	437.196	437.197	0.67	437.19(100), 287.12(12.32), 149.06(7.12), 313.14(2.52)	Isokurarinone*
**33**	37.0	$C_{25}H_{28}O_6$	423.180	423.181	0.94	423.18(85.06), 261.14(100), 161.02(54.36), 192.07(9.83)	Kushenol E/Kushenol F
**34**	37.5	$C_{20}H_{20}O_4$	323.128	323.129	0.81	323.12(100), 265.14(2.93), 164.01(2.80), 219.06(2.14)	Isoglabranin
**35**	38.2	$C_{30}H_{36}O_7$	507.238	507.239	0.95	507.23(100), 329.21(25.66), 177.01(11.87), 149.02(4.61), 205.08(3.42)	Kushenol M
**36**	38.4	$C_{25}H_{28}O_5$	407.185	407.186	0.89	407.18(100), 261.14(79.11), 339.19(12.28), 137.02(6.81)	Sophoraflavanone A
**37**	38.5	$C_{25}H_{28}O_6$	423.180	423.181	0.88	423.18(100), 339.19(34.54), 293.17(26.71), 261.15(21,20), 161.02(54.72)	Kenusanone A
**38**	38.6	$C_{25}H_{28}O_7$	439.175	439.176	0.99	439.17(18.31), 163.00(100), 135.00(23.37), 329.13(9.93), 275.16(9.21)	Sophoraflavanone D/Exiguaflavanone C

**39**	40.2	$C_{30}H_{36}O_6$	491.243	491.244	0.84	491.24(100), 329.21(93.08), 161.02(68.68), 205.08(10.34)	Kushenol B

* Identified by chromatogram of reference substance

Table S8. The energies of kurarinone/[C_8mim]BF_4 system, kurarinone, and [C_8mim]BF_4,

	Energy	Binding energy /eV
[C_8mim]BF_4	-1005.16	
Kurarinone	-1460.26	
Kurarinone/[C_8mim]BF_4	-2465.48	-1.8742248
